# Machine learning for the prediction of acute kidney injury post cardiac surgery: a systematic review and meta-analysis

**DOI:** 10.1186/s12911-026-03358-8

**Published:** 2026-03-03

**Authors:** Raja Ahsan Aftab, Zirwa Asim Butt, Baharudin Ibrahim, Lim Soo Kun

**Affiliations:** 1 Department of Clinical Pharmacy and Pharmacy Practice, Faculty of Pharmacy, Universiti Malaya, Kuala Lumpur, 50603 Malaysia; 2 School of Health, Science and Society, University of Suffolk, Ipswich, United Kingdom; 3 Department of Medicine, Faculty of Medicine, Universiti Malaya, Kuala Lumpur, 50603 Malaysia

**Keywords:** Acute kidney injury, Cardiac surgery, Machine learning, Systematic review, Meta analysis, Prediction

## Abstract

**Background and objectives:**

Cardiac surgery associated acute kidney injury can lead to increased morbidity, mortality, and hospitalization. The available risk assessment tools have limited predictive ability. Machine learning has been increasingly utilized to predict acute kidney injury in cardiac surgery patients in recent times due to its ability to handle complex clinical data. However, its predictive value remains uncertain. This study evaluates the predictive performance of machine learning models for acute kidney injury post-cardiac surgery.

**Methods:**

A systematic review and meta-analysis was conducted by searching Web of Science, PubMed, Science Direct, Google Scholar, Scopus, and Cochrane Library up to 31st December 2025. PRISMA guidelines were followed. Included studies were assessed for machine learning model performance and acute kidney injury predictors, with effect measures including area under the receiver operator characteristic curve (AUC), sensitivity, and specificity. Pooled estimates were calculated using a random-effects model with 95% confidence intervals. Risk of bias was assessed using PROBAST. The meta-analysis in our study was performed using R version 4.5.0.

**Results:**

The systematic search yielded 45 studies that met our inclusion criteria, encompassing 13 distinct model types, which include 81 models for training and 162 for validation. The overall pooled AUC was 0.83 (95% CI: 0.79–0.85) in the training and 0.76 (95% CI: 0.75–0.78) in the validation cohorts. Pooled sensitivity and specificity in the training dataset were 0.75 (95% CI: 0.71–0.79) and 0.81 (95% CI: 0.72–0.87), respectively. In the validation dataset, pooled sensitivity was 0.61 (95% CI: 0.53–0.69), while specificity was 0.82 (95% CI: 0.77–0.86). Analysis showed an overall 44.4% high risk of bias, particularly due to the analysis domain of PROBAST.

**Conclusion:**

This study suggests that machine learning based models could potentially serve as a viable framework for predicting the risk of post-cardiac surgery AKI, however, highlighting the need for model optimization and validation in a diverse population before clinical implementation.

**Trial registration:**

The study was registered with PROSPERO (CRD42024576556).

**Supplementary Information:**

The online version contains supplementary material available at 10.1186/s12911-026-03358-8.

## Introduction

Acute Kidney Injury (AKI) following cardiac surgery presents as a serious complication with poor prognosis and increased risk of mortality [1]. It is associated with a multifaceted array of exposures like cardiopulmonary bypass (CPB), tissue injury, cardiac insufficiency, and hemolysis, etc. The incidence rate of AKI associated with cardiac surgery ranges between 7% and 40% [2]. The pathophysiology of cardiac surgery-associated AKI is multifactorial and remains incompletely understood. Hypoperfusion, ischemia-reperfusion injury, neurohormonal activation, inflammation, nephrotoxin exposure, and CPB related non-pulsatile perfusion are known to be contributing factors [3]. Early prediction and interventions can be pivotal in mitigating adverse outcomes.

The clinical management of post-cardiac surgery AKI remains reactive rather than preventive. Standard Kidney Disease Improving Global Outcomes (KDIGO) criteria rely on serum creatinine, which is a delayed kidney marker, often rising only when significant loss of glomerular filtration occurs. While electronic alert systems have been implemented to improve recognition, a recent meta-analysis suggests that they have failed to consistently improve patient survival [4]. This highlights the critical need for proactive risk stratification of AKI. Recent literature indicates that early interventions using AKI care bundles can enhance kidney outcomes, but their effectiveness relies on identifying high-risk patients during a critical ‘golden window’ prior to clinical injury [5]. Hence, automated risk stratification along with targeted biomarker assessment can lead to reduced morbidity and need for continuous renal replacement therapy [6].

Machine learning (ML) offers a promising solution for the early risk stratification of AKI by using multidimensional data, surpassing conventional methods. Recent advancements in ML have equipped clinical settings with effective tools for improving AKI risk prediction. These ML algorithms enhance traditional predictive models by offering greater accuracy and the ability to handle complex, multi-dimensional datasets [7]. These predictive models can encompass a range of factors, both preoperative, intraoperative, and postoperative, to anticipate the likelihood of AKI with greater accuracy [8].

Several ML models, including random forest (RF), extreme gradient boosting (XGBoost), logistic regression (LR), artificial neural networks (ANN), and support vector machine (SVM), have been utilized to predict cardiac surgery-associated AKI and have demonstrated better accuracy in risk prediction in comparison to the traditional statistical measures, making them particularly suitable for clinical applications [9]. Understanding various ML algorithms for the prediction of AKI and their comparative efficacy is essential for risk stratification, informed decision-making, and successful implementation of interventions to prevent AKI after cardiac surgeries.

Despite the development of numerous ML models for predicting AKI, their predictive accuracy remains inconsistent. Therefore, this review aims to comprehensively evaluate the predictive capabilities of a wide range of models, particularly in the context of AKI following cardiac surgery. Understanding these variations is crucial for improving model reliability and clinical application.

## Methods

### Overview

All the articles related to the prediction of cardiac surgery-associated AKI were systematically identified, which were published in online databases as scientific literature. This systematic review and meta-analysis strictly adhered Preferred Reporting Items for Systematic Review and Meta-analysis guidelines (PRISMA). Moreover, the checklist adhering to PRISMA guidelines is provided in the *Supplementary file*. The protocol for this review has also been registered with PROSPERO [CRD42024576556].

### Search strategy

PubMed, Google Scholar, Scopus, Web of Science, Science direct and Cochrane database were searched from inception to 31st Dec, 2025. The search utilized a combination of medical subject headings (MeSH) and text/keywords related to *Acute Kidney Injury*,* Cardiac Surgery*,* and Machine learning*. Search queries with full synonym search terms are given in the Supplementary file Table S1.

### Study eligibility criteria

Only studies published in English were included, specifically quantitative studies such as randomized controlled trials, case-control studies, and cohort studies. The focus was on research involving ML models designed to predict AKI after cardiac surgery. Studies reporting the performance metrics of ML models were also included. Reviews, abstracts, letters, scientific correspondence, posters, animal studies, case reports, advertisements, theses, opinions, and editorials were excluded due to insufficient methodological data and performance metrics required for robust risk of bias assessment and quantitative meta-analysis. A detailed inclusion and exclusion criteria is given in the *Supplementary file*.

### Literature search

**Fig. 1:**
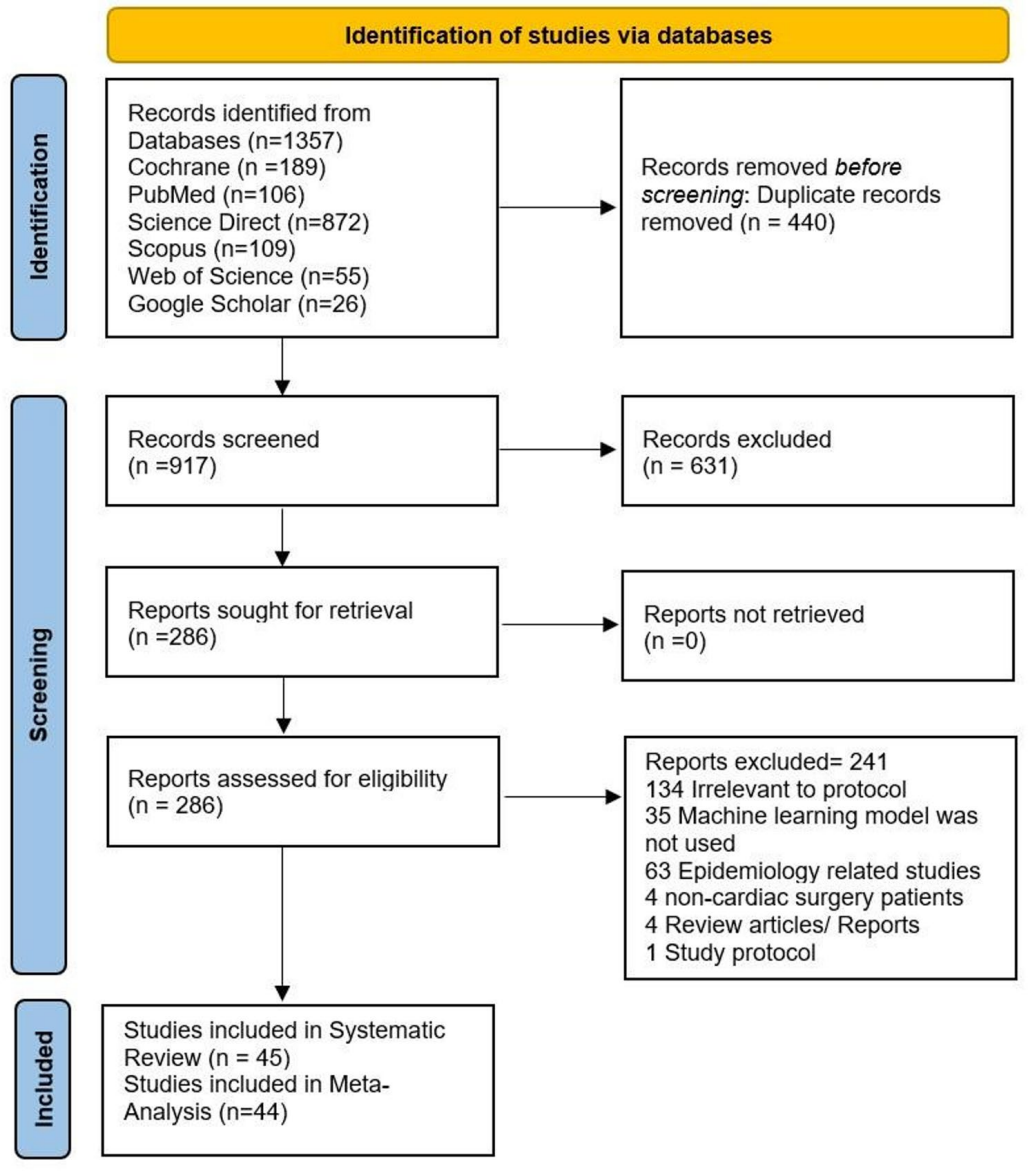
PRISMA flow diagram of the study selection process. Flow diagram illustrating the identification, screening, eligibility assessment, and inclusion of studies in the systematic review and meta-analysis in accordance with the PRISMA guidelines

The search strategy was developed following the study protocol, targeting articles that met the predefined inclusion criteria. After retrieving the articles, duplicate entries were removed using EndNote 21. A preliminary screening of titles and abstracts was conducted to filter studies related to the protocol of our study. Full-text articles of potentially relevant studies were retrieved and carefully reviewed to determine eligibility by two authors independently. The articles for inclusion and exclusion, with any discrepancies, were resolved through discussion among other authors. Articles lacking critical information necessary for the analysis were excluded from the review. The process of article selection, including the number of articles at each stage of the review, is illustrated in the PRISMA flow chart shown in Fig. 1.

### Data extraction

Data extraction focused on gathering key information from each included study based on a predefined data extraction form (Supplementary file Table S2). Two authors independently extracted data from the included studies. This process involved collecting details such as the first author, publication year, study design, sources of patient data, AKI diagnostic criteria, total number of AKI cases, and overall sample size. Additionally, we recorded the specific ML models used, the number of AKI cases, the sample size in both the training and validation datasets, methods applied to prevent overfitting, strategies for handling missing data, and approaches for variable selection and feature screening.

Performance metrics such as the Area Under the Curve (AUC), sensitivity, and specificity were also documented. The definition of AKI followed the criteria used in each study, such as KDIGO [10] or AKIN [11]. When studies did not report necessary data, we calculated it manually using available information. Studies lacking essential data were excluded from the meta-analysis but were included in the systematic review. Studies which were considered for inclusion but were excluded for protocol deviations are listed in the Supplementary file Table S3. This inclusion and exclusion of data was finalized upon mutual agreement between authors.

### Risk of bias assessment

We assessed the potential for bias in the studies included in our analysis using the PROBAST tool for risk of bias assessment (Supplementary file Table S4). It evaluates four main domains: participants, predictors, outcomes, and analysis methods. Each domain has a specific number of questions: two, three, six, and nine, respectively, that collectively determine the study’s risk of bias and overall reliability. Each question provides three possible responses: “yes/probably yes,” “no/probably no,” and “no information.” If any question within a domain is answered with “no” or “probably no,” the domain is classified as high risk. Conversely, if all questions are answered with “yes” or “probably yes,” the domain is rated as low risk.

### Quality of evidence

The Grading of Recommendations Assessment, Development and Evaluation (GRADE) methodology was used to assess the quality of evidence of all the outcomes of our study. Outcomes included pooled training and validation AUC, sensitivity, and specificity of ML models predicting AKI after cardiac surgery. Due to the observational nature of all the included studies, evidence started at low and was downgraded based on the risk of bias (PROBAST), inconsistency (high heterogeneity), indirectness (training cohorts vs. independent validation), and imprecision (wide confidence intervals). Final certainty ratings were classified as high, moderate, low, or very low.

### Data analysis

We conducted a detailed statistical analysis to evaluate the predictive performance of the machine learning models included in this study. For models that were validated in more than two independent datasets, excluding the model development set, a random-effects meta-analysis was carried out to estimate their overall performance and accuracy. This analysis also included models that were internally validated through methods such as bootstrapping or cross-validation and externally validated in at least two independent datasets.

The discriminative ability of the models was assessed by extracting reported AUC. In instances where the AUC did not have a reported 95% confidence interval (CI) or standard error, we estimated the standard error using a formula based on the number of events and participants, following the guidelines proposed by Debray et al. [12].Due to expected clinical, methodological, and algorithmic differences from variations in predictor selection, outcome definitions, and machine learning techniques, we derived pooled estimates of AUC using a random effect model with logit-transformation and restricted maximum likelihood (REML) estimation. Additionally, sensitivity and specificity were extracted from included studies, and their meta-analysis was performed using a bivariate random effect model to account for the inherent correlation between sensitivity and specificity. This model uses logit-transformed values to include both within-study and between-study variability. When primary studies did not report 2 × 2 contingency tables, true positives (TP), false positives (FP), false negatives (FN), and true negatives (TN) were generated using reported sensitivity, specificity, total sample size, and number of outcome events. These reconstructed counts were then used for bivariate random effects meta-analysis following the Reitsma model. Results were stratified by cohort (training and validation) and by ML model architecture type.

Heterogeneity was assessed using Cochran’s Q statistic and I² statistic. Heterogeneity statistics were calculated overall and within subgroups defined by ML model type. The significance of heterogeneity was evaluated using the Q test. For the Q test, a p-value less than 0.10 indicated a significant presence of heterogeneity. The I² statistics quantified the proportion of total variation across studies that was due to heterogeneity instead of sampling error. Values of 25%, 50%, and 75% represented low, moderate, and high heterogeneity, respectively.

Moreover, publication bias and small study effects were assessed among validated prediction models using the funnel plot and Egger’s test. For this, the reported primary model per study was included to ensure the statistical independence, using the AUC and its standard error derived from the 95% confidence interval. Funnel plot for visualization of asymmetry and Egger’s regression test was applied to formally evaluate small study effects.

All statistical analyses and visualizations were performed using R (version 4.5.0). The “meta” package was used to perform meta-analysis of AUC, while, “mada” package was employed to execute bivariate random effect meta-analysis for sensitivity and specificity. Data preparation, subgroup filtering, and high-resolution forest plots were generated using “readxl”, “dplyr”, “forestplot”, “magick”, and “grid” packages.

## Results

### Study selection

The databases search yielded initially 1357 articles, including Cochrane (189), PubMed (106), Science Direct (872), Scopus (109), Web of Science (55), and Google Scholar (26). After duplicate removal of 440 articles, the remaining were screened for titles and abstracts. After a thorough screening process, ultimately 45 research articles were included in the review. The PRISMA flow diagram for the study selection process is given in Fig. 1.

### Characteristics of included studies

The studies included in this systematic review and meta-analysis examined 13 distinct machine learning models applied to 401,579 cardiac surgery patients. These models included Logistic Regression (LR), Random Forest (RF), Support Vector Machine (SVM), eXtreme Gradient Boosting (XGBoost), LightGBM, k-Nearest Neighbors (k-NN), Naive Bayes (NB), Artificial Neural Networks (ANN), Ensemble methods, Decision Tree (DT), Adaptive boosting (AdaBoost), Bayesian Model Network, SoftMax, and other Gradient Boosting Decision Tree (GBDT) models.

**Fig. 2:**
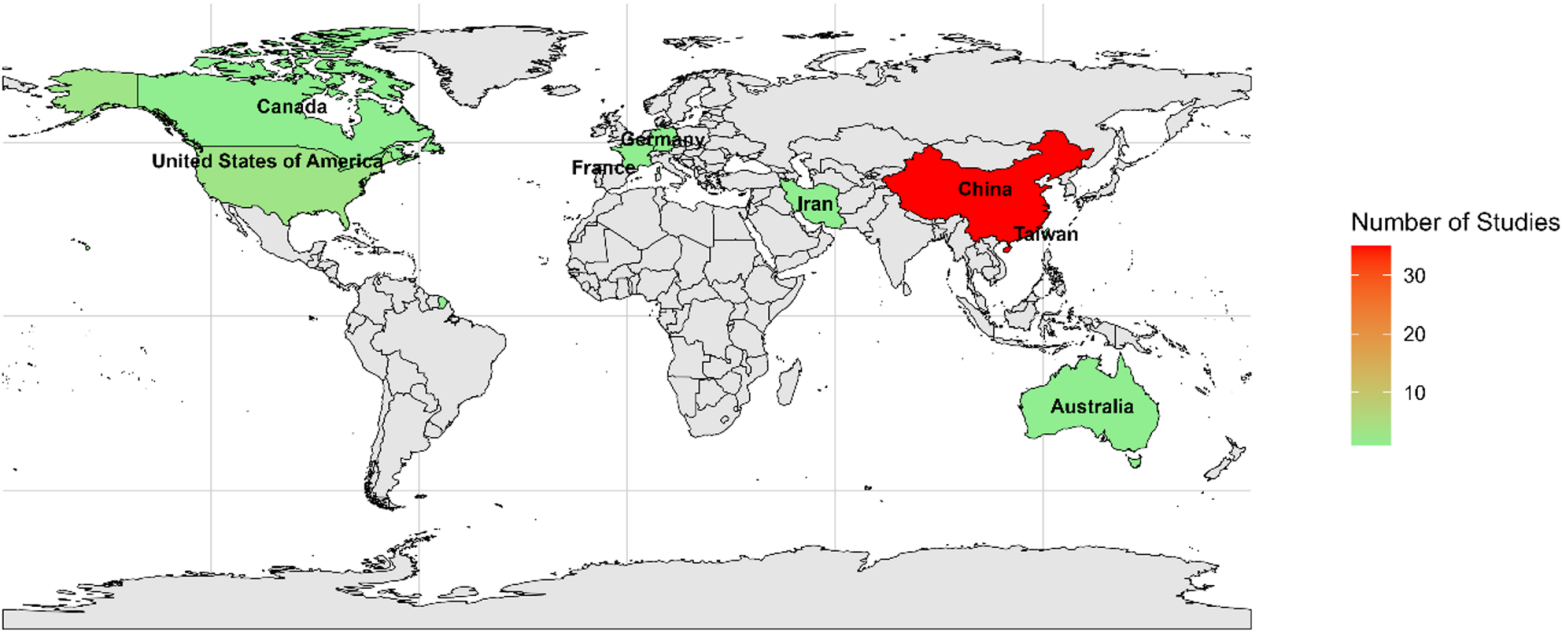
Geographic distribution of studies included in the systematic review and meta-analysis

The studies comprised 4 prospective cohort studies, 38 retrospective cohort studies, 1 retrospective case control, 1 post-hoc RCT analysis and 1 retrospective/prospective multi-center cohort study. These were retrieved from six major databases. The countries represented in the included literature were predominantly China (35 studies), followed by the United States (3 studies), Iran, Korea, Australia, Germany, France, Canada, and Taiwan, each contributing one study (Fig. 2). The detailed description of the characteristics of the included studies is given in Table 1.

**Table 1:** Characteristics of included studies

Authors	Country	Study type	Cardiac surgery types	Participants	Training data set	Validation data set	Diagnostic criteria for AKI	AKI cases	Generation way of verification set	External validation	Missing data handling	Machine learning models used
Mathieu Legrand (2013) [13]	France	Retrospective cohort	CPB for endocarditis	202	202	202	AKIN	120	V-fold CV	No	Imputation	Ensemble
Zhongli Chen (2020) [14]	China	Prospective cohort	ATAAD, TBAAD	402	204	198	AKIN	103, severe AKI 87	Two separate cohorts for screening and validation	No	Complete case analysis	LR
Yang Li (2020) [15]	China	Prospective cohort	CABG, Valvular, Aortic, and combination of these	5,533	3,639	1,894	KDIGO	2075	Temporal split, 10-fold CV	Yes	Complete case analysis	BN, LR
Guiyu Lei (2020) [16]	China	Retrospective cohort	Aortic arch	897	627	270	KDIGO	652	Random split (70:30), 10-fold CV	No	Complete case analysis	LR, RF, SVM, LightGBM
Po-Yu Tseng (2020) [17]	Taiwan	Retrospective cohort	CABG, Valvular, Aortic, and others using CPB	671	469	202	KDIGO	163	Random split (70:30)	No	Mean or median imputation	LR, DT, SVM, RF, XGBoost, Ensemble
Tim Coulson (2021) [18]	Australia	Retrospective cohort	CABG, Valve, Aortic, and others	22,731	17,048	5,683	KDIGO	5829	Random split (75:25)	No	Complete case analysis	LR
Penghua Hu (2021) [19]	China	Retrospective cohort	Valve, CABG with CPB	848	597	251	KDIGO	524	Random split, bootstrapping (*n* = 1000)	No	Multiple imputation	LR
Hao Cui (2021) [20]	China	Prospective cohort	CABG, Valve, Aorta, and others	214	95	119	AKIN	122	Clinical cohort split (CABG + VR vs. AA)	Yes	Complete case analysis	RF, SVM, LR
Penghua Hu (2021) [21]	China	Retrospective cohort	CABG, Valve, Aortic, CHD, and others	22,348	15,701	6,647	KDIGO	5,576	10-fold cross-validation, Random split	No	Multiple imputation	LR
Xin Xue (2022) [22]	China	Prospective cohort	Valve replacement, CABG, large vessel surgery, combination of these, congenital heart disease corrections	135	108	27	KDIGO	44	Random split (80:20), 5-fold CV	No	NR	LR, RF, XGBoost, SVM
Li Xinsai (2022) [23]	China	Retrospective cohort	TAAAD, TBAAD	456	319	137	KDIGO	201	Random split (70:30), 5-fold CV, bootstrapping (*n* = 1000)	No	Complete case analysis	LR, DT, XGBoost, LightGBM, RF
Hang Zhang (2022) [24]	China	Retrospective cohort	CABG, valve, and combination	1,457	1,170	287	KDIGO	353	Bootstrapping (*n* = 1000)	No	Multiple imputation	RF, Deep Forest, XGBoost, LR
Jurij Matija Kalisnik (2022) [25]	Germany	Retrospective cohort	Cardiac surgery with CPB	7,507	6,756	751	KDIGO	1,699	Random split (90:10), 10-fold CV	No	Complete case analysis	LR, XGBoost
Arman Kilic (2022) [26]	United States	Retrospective cohort	Aortic valve replacement	243,142	194,513	48,629	KDIGO	6,911	Random split (80:20), bootstrapping (*n* = 1000)	No	Complete case analysis	XGBoost
Jizhang Li (2022) [27]	China	Retrospective cohort	Acute aortic syndrome	1,637	1,318	319	KDIGO	NR	10-fold CV	Yes (319)	k-NN imputation	XGBoost, LR
Charat Thongprayoon (2022) [28]	USA	Retrospective cohort	CABG, Valve, Heart transplant, Pericardiectomy	13,158	9,244	3,914	KDIGO	4,745	Hold out validation, random split (70:15:15)	No	Multiple imputation using RF	Ensemble, RF, DT, XGBoost, ANN, LR
Hang Zhang (2022) [29]	China	Retrospective cohort	CABG, Valve, and combination of these	11,740	8,197	3,543	KDIGO	3,237	Random split (70:30), 10-fold CV	No	Multiple imputation	LR
Azar Ejmalian (2022) [30]	Iran	Retrospectiv/Prospective cohort	CABG, Valvular, Transplant, Aortic	1,435	1,148	287	KDIGO	672 1st post operative day, 413 on 7th day	Random split (80:20), 10-fold CV	No	Multiple/Iterative imputation, k-NN, and Exclusion	LR, RF, DT, XGBoost, SVM, MLP, AdaBoost
Yelena Petrosyan (2022) [31]	Canada	Retrospective cohort	CABG, Single valve, and combination of these	6,522	4,566	1,956	KDIGO	1,760	Random split (70:30)	No	NR	Ensemble, LR
Tianchen Jia (2023) [32]	China	Retrospective cohort	CABG	2,780	2,224	556	KDIGO	510	Random split (80:20)	Yes (2051)	NR	LightGBM, SVM, RF, Softmax
Yun Yan (2023) [33]	China	Retrospective cohort	Valvular surgery	3,392	2,374	1,018	KDIGO	1,713	Bootstrapping (*n* = 1000)	No	Imputation	LR, RF, XGBoost
Jiakang Shao (2023) [34]	China	Retrospective cohort	Cardiac surgery	2,108	1,686	422	KDIGO	637	5-fold CV, Cohort based split	Yes (422)	Complete case analysis	RF, ANN, LR, SVM, DT, k-NN
Yefeng Tong (2023) [35]	China	Retrospective case control	On-pump CABG	1,264	884	380	KDIGO	372	Random split (70:30), 10-fold CV	Yes	Handled by ML algorithms	XGBoost, LR, LightGBM, RF, k-NN, AdaBoost
Sai Zheng (2023) [36]	China	Retrospective cohort	Off-pump CABG	477	334	143	KDIGO	88	Random split (70:30)	No	Mean imputation	SVM, RF, GBDT, XGBoost, DT
Qian Li (2023) [37]	China	Post-hoc RCT analysis*	Cardiac surgery with CPB	2,416	1933	483	KDIGO	1,819	Random split (80:20), 5-fold CV	Yes (562, 3517)	Mean and mode imputation	LR, SVM, k-NN, DT, RF, NB, GBDT, XGBoost, LightGBM, CatBoost, AdaBoost, Extra Trees
Jicheng Jiang (2023) [38]	China	Retrospective cohort	CABG, Valve, and others	2,310	1,848	462	KDIGO	1,020	Random split (70:30)	No	Multiple imputation	LR, DT, RF, GBDT, NB, MLP
Rui Fan (2023) [39]	China	Retrospective cohort	CABG, Valve, and combination of these	778	452	326	KDIGO	210	10-fold CV	No	Multiple imputation	DF, RF, XGBoost
Anran Dai (2023) [40]	China	Retrospective cohort	Acute Aortic dissection	265	212	53	KDIGO	191	Random split (80:20), 10-fold CV	No	k-NN imputation	LR, XGBoost, RF, SVM
Yuchen Gao (2023) [41]	China	Retrospective cohort	CABG, Valve, Aortic, CHD, Cardiomyopathy, Cardiac tumour	15,880	11,117	4763	KDIGO	4845	Random split (70:30), 5-fold CV	No	Multiple imputation	XGBoost, LR
Xuejian Hou (2024) [42]	China	Retrospective cohort	CABG	1,767	1,414	353	KDIGO	417	Random split (80:20), 5-fold repetition	No	NR	RF, LightGBM, XGBoost, LR, NB
Zhihe Zeng (2024) [43]	China	Retrospective cohort	Off-pump CABG	1,110	777	333	KDIGO	642	Random split (70:30), 5-fold CV	No	Deep learning-based imputation	LR, RF, DT, XGBoost
Xiaolong Liu (2024) [44]	China	Retrospective cohort	Total Aortic arch replacement	572	394	178	KDIGO	131	Random split (70:30), 10-fold CV, bootstrapping (*n* = 100)	No	NR	ANN, LR
Yuanhan Chen (2024) [45]	China	Retrospective cohort	On-pump, Valve, Aortic, CHD, combination of these and others	5,368	3,768	1,600	KDIGO	2681	Random split (70:30)	No	Multiple imputation	LR
Changho HAN (2024) [46]	Korea	Retrospective cohort	Cardiac surgery with CPB	2,003	1,402	601	KDIGO	322	Random split (70:30)	No	NR	XGBoost
Yuezi Song (2024) [47]	China	Retrospective cohort	Off-pump CABG	701	561	140	KDIGO	73	Random split (80:20)	No	Complete case analysis (Excluded)	LR, GBDT, XGBoost, AdaBoost, RF, SVM, k-NN, DT
XinPei Liu (2024) [48]	China	Retrospective cohort	Active infective endocarditis cardiac surgery	527	368	159	KDIGO	261	Random split (70:30)	No	Mean or median imputation	LR, Linear SVM, Radial SVM, XGBoost, DT, RF
Yang Zhang (2025) [49]	China	Retrospective cohort	CABG	520	364	156	KDIGO	135	Random split (70:30), Grid search CV	No	k-NN imputation	RF, XGBoost, LR, LightGBM, Softmax, SVM
Kuroush Nezafati (2025) [50]	USA	Retrospective cohort	CABG, Valvular, Aortic, Septal myectomy, and combination using CPB	602	481	121	KDIGO	110	Random split (80:20), 5-fold Grid search CV	No	Complete case analysis excluded	RF, LSTM
Ling Chen (2025) [51]	China	Retrospective cohort	ATAAD	1,010	758	252	KDIGO	466	Random split (75:25)	Yes (201)	Complete case analysis excluded	LR
Haiming Li (2025) [52]	China	Retrospective cohort	CABG	2,155	1,509	646	KDIGO	365	Random split (70:30)	No	Mean imputation (< 20% missing), otherwise excluded	LR, DT, RF, SVM, XGBoost, AdaBoost, GBDT, LightGBM, k-NN
Biao Hou (2025) [53]	China	Retrospective cohort	CABG	3,043	2,130	913	KDIGO	465	Random split (70:30)	Yes (878)	Complete case analysis excluded	SVM, DT, RF, AdaBoost, XGBoost
Zishan Li (2025) [54]	China	Retrospective cohort	Cardiac surgery	4,565	3,196	1,369	KDIGO	3,046	Random split (70:30), 10-fold CV	No	Multiple imputation	RF, LASSO regression
Yang Xu (2025) [55]	China	Retrospective cohort	CABG, Valve, and combination of these	1,304	916	388	KDIGO	1,028	Random split (70:30), 10-fold CV	No	Multiple imputation (< 20% missing)	LR, LASSO regression, RF
Zheyuan Chen (2025) [56]	China	Retrospective cohort	ATAAD	1,350	1,148	202	KDIGO	586	Random split (85:15), 10-fold CV	No	Multiple imputation	GBDT, LightGBM, RF, k-NN, ANN, NB, LR
Qin Sun (2025) [57]	China	Retrospective cohort	CABG, Valve or combination of these	2,277	1,593	684	KDIGO	514	Random split (70:30)	Yes (1174)	missForest imputation	LR, RF, GBDT, XGBoost, SVM, Ensemble

Abbreviations: AKIN, Acute Kidney Injury Network; KDIGO, Kidney Disease: Improving Global Outcomes; ATAAD, Acute Type A Aortic Dissection; CABG, Coronary Artery Bypass Grafting; CHD, Congenital Heart Disease; CPB, Cardiopulmonary Bypass; RCT, Randomized Controlled Trial; TAAAD, Total Acute Aortic Arch Dissection; TBAAD, Type B Acute Aortic Dissection. AdaBoost, Adaptive Boosting; ANN, Artificial Neural Network; BMA, Bayesian Model Averaging; BN, Bayesian Network; CatBoost, Categorical Boosting; DF, Deep Forest; DT, Decision Tree; GBDT, Gradient Boosting Decision Tree; k-NN, k-Nearest Neighbors; LASSO, Least Absolute Shrinkage and Selection Operator; LightGBM, Light Gradient Boosting Machine; LR, Logistic Regression; LSTM, Long Short-Term Memory; MLP, Multilayer Perceptron; NB, Naïve Bayes; RF, Random Forest; SVM, Support Vector Machine; XGBoost, Extreme Gradient Boosting

### Risk of bias assessment

**Fig. 3:**
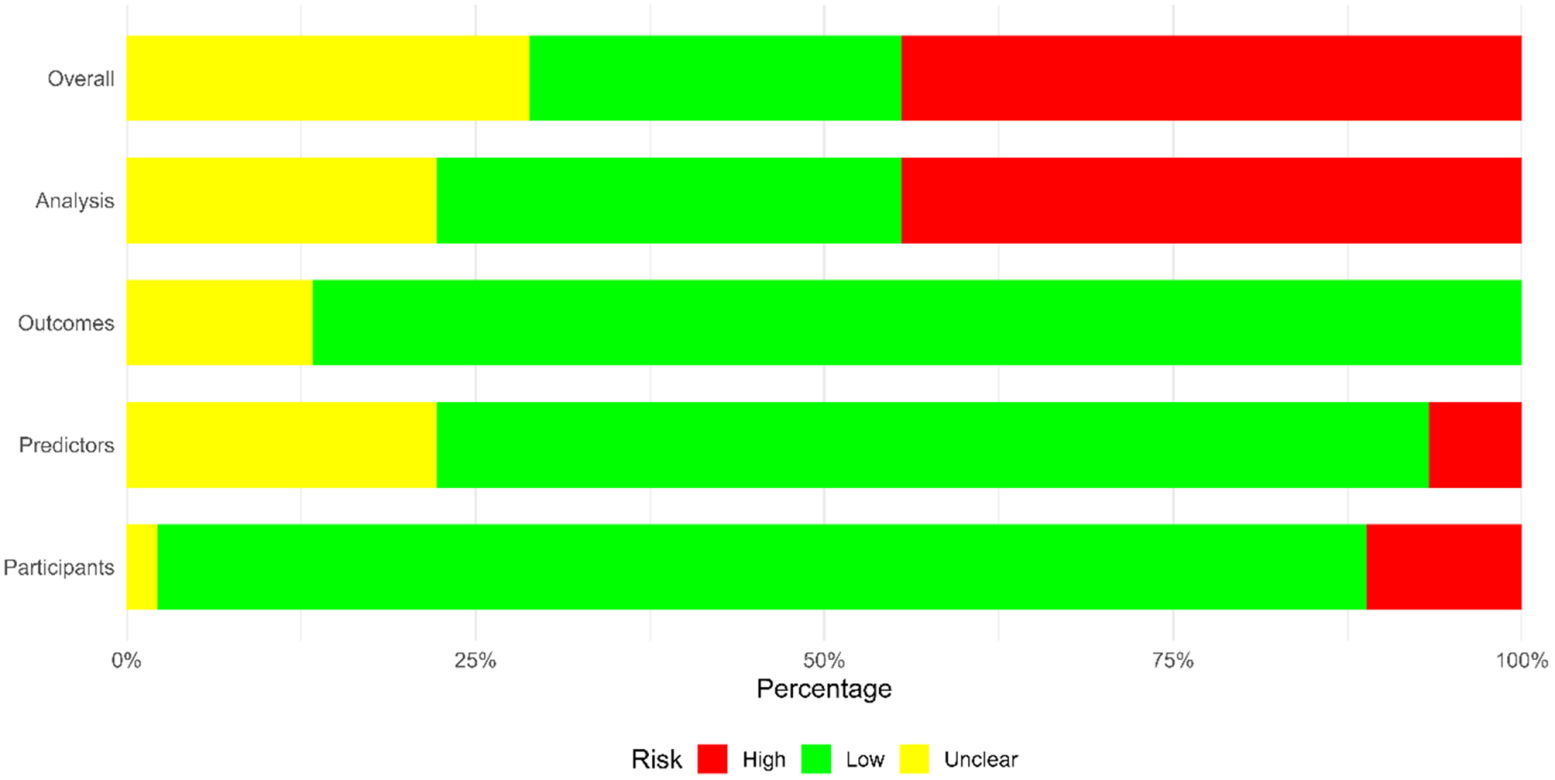
Risk of bias assessment results using the PROBAST tool

The quality assessment of the included studies was evaluated using the PROBAST tool. The domain-specific assessment is provided in Fig. 3, while detailed individual study ratings are available in the Supplementary file Table S5. Approximately 26.7% of the included studies were classified as having a low overall risk of bias, 44.4% were rated as high risk, and the remaining 28.9% were categorized as unclear. The participant domain showed a relatively small proportion of high risk, with 86.7% of studies rated as low risk and only 11.1% rated as high risk. Regarding the predictors domain, low risk of bias was observed in 71.1% of the studies, while 22.2% were rated as unclear. In the outcome domain, assessment of AKI outcome was rated as low risk in 86.7% of the studies, with the remaining 13.3% classified as unclear. However, the analysis domain raised more concern, with 44.4% of studies being classified as having a high risk of bias, whereas 33.3% of studies were rated as low risk, and 22.2% were unclear (Fig. 3).

### AUC

**Fig. 4:**
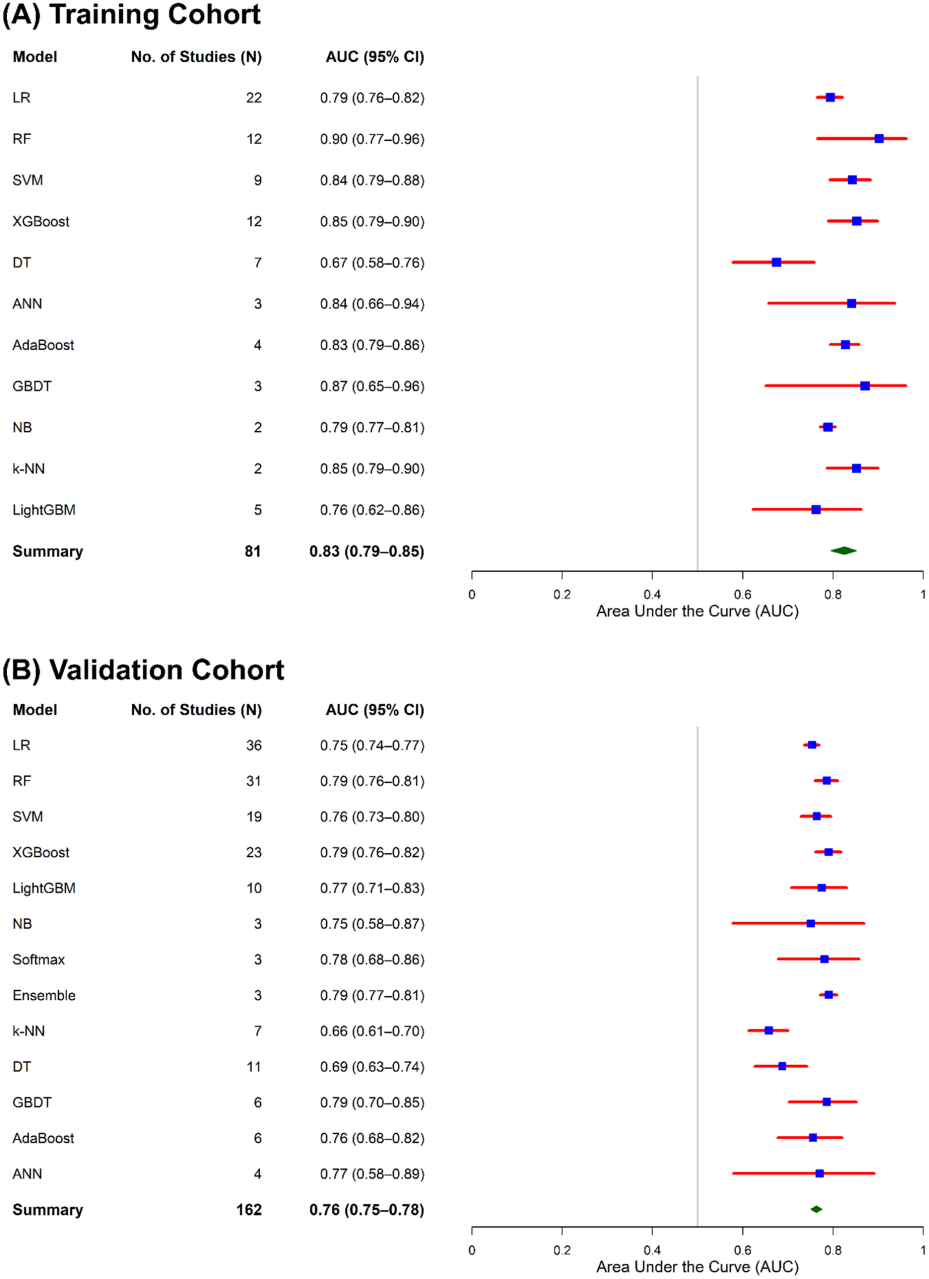
Forest plots of pooled AUCs for machine learning models predicting AKI: training (**A**), validation (**B**)

Figure 4 illustrates that a total of 81 models were included in the meta-analysis of AUC in the training cohort for the prediction of AKI after cardiac surgery, while 162 models were included in the validation cohort derived from 44 primary studies included in the meta-analysis. Using random effect meta-analysis, the pooled AUC was estimated to be 0.83 (95% CI: 0.79–0.85) in the training cohort and 0.76 (95% CI: 0.75–0.78) in the validation cohort. Subgroup analysis stratified by ML model architecture suggested significant differences in discriminative performance across model types in both cohorts. In the training cohort, RF 0.90 (95% CI: 0.77–0.96), GBDT 0.87 (95% CI: 0.65–0.96), and XGBoost 0.85 (95% CI: 0.79–0.90) exhibited comparatively higher pooled AUC estimates. In the validation cohort, XGBoost 0.79 (95% CI: 0.76–0.82), RF 0.79 (95% CI: 0.76–0.81), and Ensemble methods 0.79 (95% CI: 0.77–0.81) showed relatively higher discriminative performance compared with other models.

Substantial heterogeneity was observed across most ML model types in both training and validation cohorts. In the training cohort, the random effect meta-analysis revealed considerable heterogeneity (I^2^ =98.2%, *p* < 0.001). Similarly, the validation cohort exhibited very high heterogeneity (I^2^ =98.7%, *p* < 0.001), indicating marked variability in predictive performance across included ML approaches. Furthermore, model specific subgroup analysis revealed persistently high within subgroup heterogeneity for most algorithms (Supplementary file Table S6 and S7). Furthermore, tests for subgroup differences indicated significant between model heterogeneity in both cohorts (training Q = 26.7, p 0.0034; validation Q = 51.37 *p* < 0.001).

### Sensitivity and specificity

**Fig. 5:**
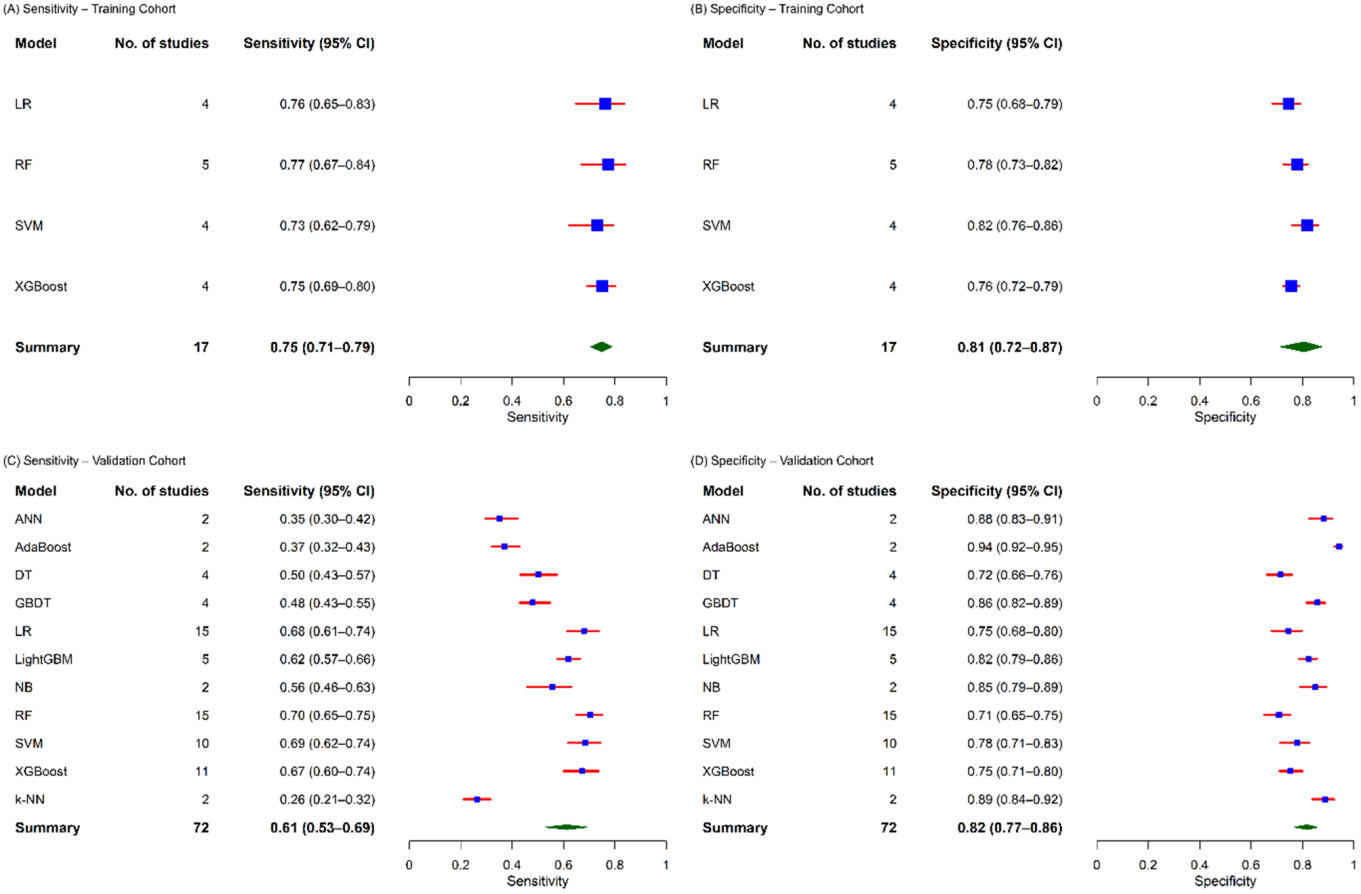
Forest plots of sensitivity and specificity for machine learning models: training (**A–B**) and validation (**C–D**) cohorts

Figure 5 illustrates a total of 17 models were evaluated in the training cohort, reporting sensitivity and specificity, which represented 7,997 patients undergoing cardiac surgery. The pooled sensitivity across these ML models was 0.75 (95% CI: 0.71–0.79), while the pooled specificity was 0.81 (95% CI: 0.72–0.87). In the validation cohorts, 72 models reported sensitivity and specificity, encompassing 10,063 patients. In this setting, the pooled sensitivity declined to 0.61 (95% CI: 0.53–0.69), whereas the pooled specificity remained consistently high at 0.82 (95% CI: 0.77–0.86).

Bivariate random effect meta-analysis of diagnostic performance of included ML models showed moderate to substantial between-model heterogeneity, with variability evident in both training (τ = 0.42–1.04) and validation (τ = 1.27–1.44) cohorts. Detailed heterogeneity estimates are presented in the Supplementary file Table S8.

### Quality of evidence

Using the GRADE framework, the certainty of evidence for pooled estimates of AUC, sensitivity, and specificity was rated as very low across both training and validation cohorts. Detailed GRADE assessments for each outcome are presented in the supplementary file Table S9*.*

Moreover, the assessment of small study effects using Egger’s regression test did not indicate evidence of asymmetry (*p* = 0.41) (Supplementary file Figure S1).

### Predictors of acute kidney injury

**Fig. 6:**
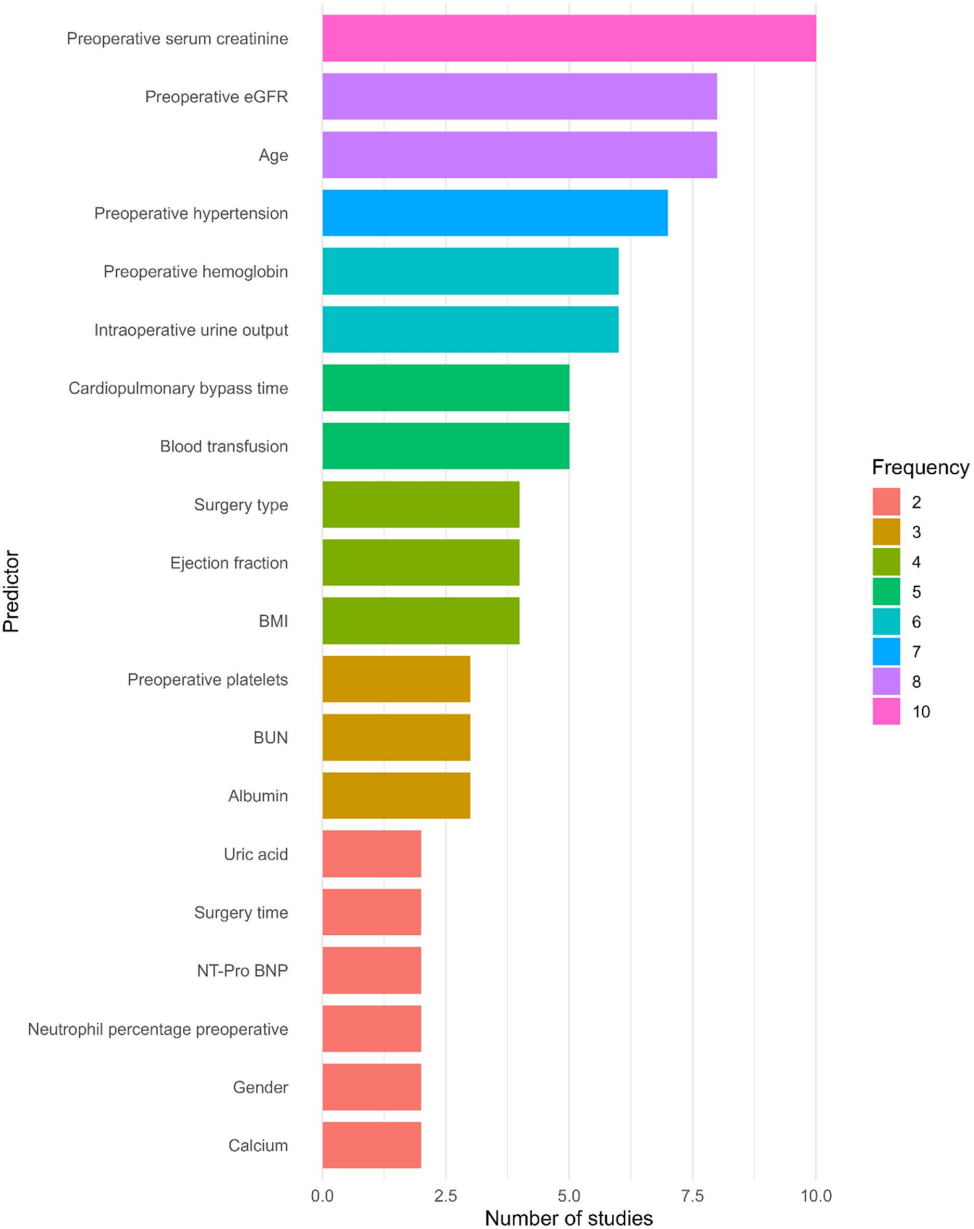
Frequently reported predictors of ML models for the prediction of AKI after cardiac surgery

Across the included studies, predictors contributing to model performance were extracted based on reported feature importance rankings. The top five features utilized by ML models reported in each study were identified and summarized in Fig. 6.

Among the most frequently reported predictors were preoperative serum creatinine, eGFR, age, and hypertension. Other predictors that appeared prominently were preoperative hemoglobin, intraoperative urine output, CPB time, and blood transfusion. A detailed list of predictors to develop ML models utilized by studies included in this review is given in the Supplementary file Table S10.

## Discussion

The use of ML for predicting AKI after cardiac surgery has surged in recent years. Despite considerable progress, systematic reviews and meta-analyses that assess the predictive performance of these models remain limited. This systematic review and meta-analysis evaluated multiple ML models and highlighted their predictive performance using AUC, sensitivity, and specificity in training and validation cohorts, offering valuable insights into their practical application in clinical settings. Across the 13 distinct models evaluated, ranging from traditional algorithms like LR to more advanced techniques such as gradient boosting (XGBoost, LightGBM) and ensemble methods, predictive outcomes were observed to have moderate to high performance. Our findings indicate that ML algorithms demonstrate good discriminative ability for AKI, with a pooled AUC of 0.83 (95% CI: 0.80–0.85) in the training and 0.76 (95% CI: 0.75–0.78) in the validation cohorts.

Risk stratification of AKI in cardiac surgery patients is usually performed with traditional risk analysis systems like regression models and risk scores. However, they have significant limitations in terms of handling complex, high-dimensional data, adapting to new information, and providing personalized, real-time predictions [22]. These shortcomings make them less suited for modern, data-rich healthcare environments, where ML models offer improved flexibility, accuracy, and predictive power [58].

The more advanced models, like ensemble techniques, RF, gradient boosting achieved the higher pooled AUCs in this study, whereas simpler approaches like DT showed lower discrimination. Ensemble-based approaches benefit from their ability to combine predictions from multiple weak learners, effectively reducing variance and overfitting [59]. Gradient boosting methods iteratively correct misclassification from their previous trees, enabling them to capture complex nonlinear relationships among predictors that are common in clinical settings [60]. However, simpler models like DT suffer, as they are more prone to instability and overfitting, particularly when applied to heterogeneous clinical datasets with noisy or correlated predictors [1].

Notably, LR exhibited stable performance in both cohorts, suggesting that LR can be robust when applied to well-established clinical predictors with approximate linear associations to AKI. Conventional statistical approaches can remain clinically relevant, particularly when interpretability is crucial [61]. Moreover, the overall decrease in predictive performance of several ML models across training to validation cohorts signals potential overfitting in the training datasets, emphasizing the necessity of evaluation on independent datasets for robust prediction.

Bivariate random effect meta-analysis of sensitivity and specificity accounted for the trade-off between identifying patients at risk and avoiding false positives. In the validation cohort, LR and RF achieved moderate sensitivity (0.68–0.79) and specificity (0.71–0.78), whereas algorithms such as k-NN and ANN showed lower sensitivity. Ensemble and boosting methods generally achieved higher specificity, reflecting greater accuracy in correctly identifying patients without AKI. These trends highlight the importance of tailoring model selection to the intended clinical objective, such as prioritizing higher sensitivity to minimize missed AKI cases or specificity to reduce unnecessary interventions.

A substantial heterogeneity was observed across ML models in predicting AKI after cardiac surgery, which is common in the meta-analysis of diagnostic accuracy and ML-based prediction models. Unlike conventional intervention studies, predictive modelling research inherently varies in terms of model architecture, feature selection, dataset characteristics, and validation strategies. This between-model variance is consistent with the prior studies, which have shown that differences in patient populations, input variables, and outcome definitions can influence the predictive performance of ML models. While we did not explore the additional sources of heterogeneity due to inconsistent reporting of covariates, these findings highlight that model performance may vary depending on the study design and data characteristics, emphasizing the need for external validation before clinical implementation.

The clinical implementation of ML-based prediction faces several practical considerations. High-performing models such as Ensemble, RF, and XGBoost may offer superior accuracy but are less interpretable, potentially limiting clinician acceptance. The limitation of complex models, often referred to as “black box” models, necessitates the use of Explainable AI frameworks, such as SHapley Additive Explanation (SHAP), for a better understanding of the underlying features involved in predictions [62]. This understanding offers clinical insights regarding the specific physiological drivers behind an AKI alert. In contrast, LR and other transparent models provide interpretable outputs that can facilitate decision-making at the bedside.

Beyond algorithmic performance, the predictive accuracy of the ML models is substantially influenced by the selection of optimal modelling variables and their quality. Across the included studies, the input variables were a mix of demographic, preoperative, intraoperative, and early postoperative biomarkers. The most frequently reported features for the prediction of AKI after cardiac surgery were preoperative serum creatinine, eGFR, age, and hypertension, reflecting their well-established roles in AKI risk. Preoperative serum creatinine levels and eGFR are critical in assessing baseline renal function, and their levels often indicate pre-existing kidney damage or reduced renal reserve, placing patients at higher risk of AKI following surgery [63]. These variables also provide essential information on renal reserve and vulnerability to ischemic or inflammatory insults during cardiac surgery. Intraoperative urine output is another indicator of renal function during surgery, and its decrease is one of the earliest signs of impending AKI [64]. The prominence of renal function markers across studies suggests that they should remain a core component of any predictive model for AKI. Similarly, age is a known risk factor for AKI due to the decline in kidney function with advancing age and the increased likelihood of comorbidities in older patients [65]. Hypertension and baseline hemoglobin also emerged as important predictors of postoperative AKI across the included studies, highlighting their clinical relevance in AKI risk stratification models.

A notable finding from this review is geographical bias, with 77.7% (35/45) of studies originating from China. This concentration introduces a potential geographical bias that could limit the global applicability of the results. Healthcare systems in Southeast Asia, i.e., China, may differ significantly from the West or other regions in terms of surgical protocols, perioperative care practices, and genetic predispositions to AKI that are specific to different ethnic groups. Furthermore, the prevalence of comorbidities like diabetes and the timing of surgical procedures often vary by region. Therefore, although the ML models analyzed in this review demonstrated acceptable performance within their study populations, their generalizability remains uncertain. Broader evaluation in diverse, multi-ethnic, and international populations is required, as only a limited proportion of included studies performed external validation using large, publicly available datasets such as the US-based MIMIC database [55].

Despite the strengths of this meta-analysis, several limitations must be acknowledged. First, substantial heterogeneity was observed across the included models, likely reflecting differences in modeling strategies and validation approaches. Although a random-effect model was used to account for between-model variability, the pooled performance metrics must be considered as average estimates across heterogeneous settings rather than universally applicable benchmarks. Second, most included studies were retrospective and relied on single-center datasets, with a limited proportion of studies that conducted external validations, raising concerns about the global applicability. Third, a lack of standardization in ML techniques across studies, such as differences in hyperparameter tuning, model optimization, and handling of missing data, also complicates the interpretation and replicability of results. Finally, the effect of publication bias cannot be fully excluded, as high-performance studies have more chances to get published. Although funnel plot asymmetry and statistical tests were explored, these methods have limited power in meta-analysis of predictive modeling studies.

## Conclusion

This meta-analysis offers a comprehensive evaluation of the effectiveness of various ML models in predicting AKI after cardiac surgery. Overall, the ML models exhibited moderate to high predictive ability, with a pooled AUC indicating reliable discrimination across the studies. However, significant variability was noted, likely due to differences in modeling approaches, predictor selection, and validation strategies. Despite this heterogeneity and the associated risk of bias, our findings support the potential of ML-based prediction to assist in the risk stratification of AKI, which could help guide preventive strategies in clinical practice. Future research should prioritize external validation, standardization of reporting, and the integration of ML models into clinical workflows to evaluate their real-world effectiveness.

## Supplementary Information

Below is the link to the electronic supplementary material.

Supplementary Material 1

## Data Availability

The dataset supporting the conclusions of this article is included within the article and its additional files.

## Abbreviations

AKI: Acute kidney injury
AUC: Area under the curve
SVM: Support vector machine
ML: Machine learning
PRISMA: Preferred reporting items for systematic reviews and meta-analyses
SHAP: SHapley additive explanation
KDIGO: Kidney disease improving global outcomes
AKIN: Acute kidney injury network
RF: Random forest
LR: Logistic regression
k-NN: k-Nearest neighbours
ANN: Artificial neural networks
XGBoost: eXtreme gradient boosting
DT: Decision tree
GBDT: Gradient boosting decision tree
AdaBoost: Adaptive boosting
NB: Naive bayes
