# Supplementary material for: Machine learning for the prediction of acute kidney injury post cardiac surgery: a systematic review and meta-analysis
Source: BMC Med Inform Decis Mak. 2026 Mar 3;26:133. doi: 10.1186/s12911-026-03358-8 (PMC13101215; doi:10.1186/s12911-026-03358-8)
Supplement: Supplementary file 1 — Supplementary Material 1 [file 12911_2026_3358_MOESM1_ESM.pdf]

Supplementary Material

Inclusion Criteria

Included studies were retrospective, prospective cohort studies and case-control studies that assessed the performance of machine learning models used to predict acute kidney injury after cardiac surgery. Randomized clinical trials (RCT) were part of the search criteria but none of the qualifying studies for systematic review and meta-analysis was RCT. Systematic reviews and other relevant review articles were also used to identify any primary research articles relevant to our work. Studies written in the English language were only included in our review irrespective of country or origin of publication.

Exclusion Criteria

Systematic literature reviews, abstracts, scientific correspondence, posters, animal studies, case reports, advertisements, thesis, opinions and editorials were excluded. Studies in which extraction of data was not possible were also excluded.

Table S1. Search Strategies

Database		Search terms	# of articles
Pubmed		(("Machine learning"[tw] OR "artificial intelligence"[tw] OR "machine learning algorithm"[tw] OR "Machine Learning"[Mesh]) AND ("acute kidney injury"[tw] OR "AKI" [tw] OR "acute kidney failure"[tw] OR "acute renal failure"[tw] OR "Acute Kidney Injury"[Mesh])) AND ("cardiac surgery"[tw] OR "heart surgery"[tw] OR "thoracic surgery"[tw] OR CABG[tw] OR "cardiac surgery associated acute kidney injury"[tw] OR "Thoracic Surgical Procedures"[Mesh])	106
Google scholar		allintitle: ("machine learning" OR "artificial intelligence" OR "machine learning algorithm") AND ("Acute kidney Injury") AND ("cardiac surgery" OR "CABG" OR "Cardiac surgery associated AKI" OR "heart surgery" OR "thoracic surgery")	26
Web of Science		TS=((machine learning OR machine learnings OR "machine-learned" OR "machine-learning") OR (artificial intelligence OR AI)) AND TS=(algorithm OR algorithms) AND TS=(acute kidney injury OR AKI OR "acute renal failure" OR "acute renal insufficiency") AND TS=(cardiac surgery OR CABG OR "coronary artery bypass graft")	55
Scopus		("machine learning" OR "artificial intelligence" OR "machine learning algorithm") AND ("Acute kidney Injury") AND ("cardiac surgery" OR "CABG" OR "Cardiac surgery associated AKI" OR "heart surgery" OR "thoracic surgery")	109
Science direct		("machine learning" OR "artificial intelligence" OR "machine learning algorithm") AND ("Acute kidney Injury") AND ("cardiac surgery" OR "CABG" OR "Cardiac surgery associated AKI" OR "heart surgery" OR "thoracic surgery")	872
Cochrane library	#1	MeSH descriptor: [Thoracic Surgery] explode all trees	241
	#2	(Surgery, Thoracic):ti,ab,kw OR (Surgery, Cardiac):ti,ab,kw OR (Surgery, Heart):ti,ab,kw OR (Heart Surgery):ti,ab,kw AND (Cardiac Surgery):ti,ab,kw	58134
	#3	MeSH descriptor: [Machine Learning] explode all trees	1246
	#4	(machine learning):ti,ab,kw OR (Deep learning):ti,ab,kw OR (Prediction model):ti,ab,kw OR (Transfer Learning):ti,ab,kw OR (random forest):ti,ab,kw	13204
	#5	(artificial neural network):ti,ab,kw OR (ANN):ti,ab,kw OR (Support vector machine):ti,ab,kw OR (SVM):ti,ab,kw OR (Nomogram):ti,ab,kw	4873
	#6	(COX):ti,ab,kw OR (XGboost):ti,ab,kw OR (Logistic):ti,ab,kw OR (Decision tree):ti,ab,kw OR (c-index):ti,ab,kw	59326
	#7	(ROC):ti,ab,kw OR (AUC):ti,ab,kw OR (External validation):ti,ab,kw	31130
	#8	#1 or #2	58134
	#9	#3 or #4 or #5 or #6 or #7	97926
	#10	MeSH descriptor: [Acute Kidney Injury] explode all trees	2547
	#11	(Acute Kidney Injuries):ti,ab,kw OR (Kidney Injuries, Acute):ti,ab,kw OR (Kidney Injury, Acute):ti,ab,kw OR (Acute Renal Injury):ti,ab,kw OR (Acute Renal Injuries):ti,ab,kw	7111
	#12	(Renal Injuries, Acute):ti,ab,kw OR (Renal Injury, Acute):ti,ab,kw OR (Renal Insufficiency, Acute):ti,ab,kw OR (Acute Renal Insufficiencies):ti,ab,kw OR (Renal Insufficiencies, Acute):ti,ab,kw	5172
	#13	(Acute Renal Insufficiency):ti,ab,kw OR (Kidney Insufficiency, Acute):ti,ab,kw OR (Acute Kidney Insufficiencies):ti,ab,kw OR (Kidney Insufficiencies, Acute):ti,ab,kw OR (Acute Kidney Insufficiency):ti,ab,kw	1493
	#14	(Kidney Failure, Acute):ti,ab,kw OR (Acute Kidney Failures):ti,ab,kw OR (Kidney Failures, Acute):ti,ab,kw OR (Acute Renal Failure):ti,ab,kw OR (Acute Renal Failures):ti,ab,kw	10076
	#15	(Renal Failures, Acute):ti,ab,kw OR (Renal Failure, Acute):ti,ab,kw OR (Acute Kidney Failure):ti,ab,kw	10067
	#16	#10 or #11 or #12 or #13 or #14 or #15	13494
	#17	#8 and #9 and #16	189

Table S2. Extracted variables

Sr #	Variables
1	Authors
2	Year of Publication
3	Country
4	Type of Study
5	Total Participants
6	Training data sets
7	Validation data sets
8	Diagnostic criteria for AKI
9	Outcome
10	AKI cases
11	AUC training
12	AUC validation
13	Generation way of verification set
14	Training sensitivity and specificity
15	Validation sensitivity and specificity
16	External validation
17	Feature selection
18	Missing data handling
19	Machine learning models used
20	Cardiac surgery type

Table S3. Excluded studies that were considered for inclusion

Study	Name	Exclusion reason
Dass et al (2012)[10]	Fluid balance as an early indicator of acute kidney injury in CV surgery	No machine-learning model utilized
Datta et al (2021)[11]	FIBER: Enabling flexible retrieval of electronic health records data for clinical predictive modeling	Not focussed on our study protocol outcome
Yao et al (2021)[12]	Application of artificial intelligence in renal disease	Irrelevant protocol
Hu et al (2022)[13]	Application of interpretable machine learning for early prediction of prognosis in acute kidney injury	Non-cardiac surgery patients
Reddy et al (2023)[14]	Advanced artificial intelligence-guided hemodynamic management within cardiac enhanced recovery after surgery pathways: A multi-institution review	Not relevant to our study protocol
Schmid et al (2023)[15]	Algorithm-based detection of acute kidney injury according to full KDIGO criteria including urine output following cardiac surgery: a descriptive analysis	Performance of machine learning models was not measured

Table S4. PROBAST signalling questions for Risk of Bias Assessment

Domains	Questions
Participants	1·1 Were appropriate data sources used, e.g., cohort, RCT, or nested case-control study data?
	1·2. Were all inclusions and exclusions of participants appropriate?
Predictors	2·1. Were predictors defined and assessed in a similar way for all participants?
	2·2. Were predictor assessments made without knowledge of outcome data?
	2·3. Are all predictors available at the time the model is intended to be used?
Outcome	3·1. Was the outcome determined appropriately?
	3·2. Was a prespecified or standard outcome definition used?
	3·3. Were predictors excluded from the outcome definition?
	3·4. Was the outcome defined and determined in a similar way for all participants?
	3·5. Was the outcome determined without knowledge of predictor information?
	3·6. Was the time interval between predictor assessment and outcome determination appropriate?
Analysis	4·1 Were there a reasonable number of participants with the outcome?
	4·2. Were continuous and categorical predictors handled appropriately?
	4·3 Were all enrolled participants included in the analysis?
	4·4. Were participants with missing data handled appropriately?
	4·5. Was selection of predictors based on univariable analysis avoided?
	4·6. Were complexities in the data (e.g., censoring, competing risks, sampling of control participants) accounted for appropriately?
	4·7. Were relevant model performance measures evaluated appropriately?
	4·8. Was model overfitting, underfitting, and optimism in model performance accounted for?
	4·9. Do predictors and their assigned weights in the final model correspond to the results from the reported multivariable analysis?

Table S5. PROBAST Risk of Bias Assessment Results

**Study**	**D1 Participants**	**D2 Predictors**	**D3 Outcomes**	**D4 Analysis**	**Overall**
Mathieu Legrand (2013)	Low	Unclear	Low	High	High
Zhongli Chen (2020)	Low	Low	Yes	Low	Low
Yang Li (2020)	Low	Low	Low	Unclear	Unclear
Guiyu Lei (2020)	Low	Low	Low	Low	Low
Po-Yu Tseng (2020)	Low	Low	Low	Unclear	Unclear
Tim Coulson (2021)	Low	Low	Low	High	High
Penghua Hu (2021)	Low	Low	Low	Unclear	Unclear
Hao Cui (2021)	High	Low	Low	High	High
Penghua Hu (2021)	Low	Low	Low	Unclear	Unclear
Xin Xue (2022)	Low	Unclear	Low	Low	Unclear
Li Xinsai (2022)	Low	Unclear	Low	Unclear	Unclear
Hang Zhang (2022)	Low	Low	Low	Low	Low

Jurij Matija Kalisnik (2022)	Low	Low	Unclear	High	High
Arman Kilic (2022)	Low	Low	Low	High	High
Jizhang Li (2022)	Low	Unclear	Unclear	Low	Unclear
Charat Thongprayoon (2022)	Low	Low	Low	Low	Low
Hang Zhang (2022)	Low	Low	Low	Low	Low
Azar Ejmalian (2022)	Low	Low	Low	Unclear	Unclear
Yelena Petrosyan (2022)	Low	Low	Low	Unclear	Unclear
Tianchen Jia (2023)	Low	Low	Low	Low	Low
Yun Yan (2023)	Low	Unclear	Low	Low	Low
Jiakang Shao (2023)	Low	Unclear	Low	High	High
Yefeng Tong (2023)	Low	Low	Unclear	Unclear	Unclear
Sai Zheng (2023)	Low	Unclear	Unclear	High	High
Qian Li (2023)	Low	Unclear	Unclear	Low	Low
Jicheng Jiang (2023)	Low	Low	Unclear	High	High
Rui Fan (2023)	Low	Low	Low	Low	Low
Anran Dai (2023)	Low	Low	Low	High	High
Yuchen Gao (2023)	Low	Low	Low	Low	Low
Xuejian Hou (2024)	Low	Low	Low	High	High
Zhihe Zeng (2024)	Low	Low	Low	Low	Low
Xiaolong Liu (2024)	Low	Low	Low	Unclear	Unclear
Yuanhan Chen (2024)	Low	Low	Low	Unclear	Unclear
Changho HAN (2024)	Low	Low	Low	High	High

Yuezi Song (2024)	No	Low	Low	High	High
XinPei Liu (2024)	Low	Low	Low	Low	Low
Yang Zhang (2025)	No	No	Low	High	High
Kuroush Nezafati (2025)	No	No	Low	High	High
Ling Chen (2025)	No	Low	Low	High	High
Haiming Li (2025)	Low	Low	Low	High	High
Biao Hou (2025)	Low	Low	Low	High	High
Zishan Li (2025)	Low	High	Low	High	High
Yang Xu (2025)	Low	Unclear	Low	High	High
Zheyuan Chen (2025)	Low	Unclear	Low	High	High
Qin Sun (2025)	Unclear	Low	Low	Low	Unclear

Table S6. Summary statistics of Pooled AUC meta-analysis (training cohort)

ML architecture	No. of models (k)	Pooled logit AUC (95% CI)	τ^2	τ	I^2 (%)	Q statistic	p for heterogeneity
All models	81	1.55 (1.35–1.75)	0.78	0.88	98.2	4470.4	<0.001
Logistic regression (LR)	22	1.35 (1.18–1.52)	0.14	0.38	97.3	775.2	<0.001
Random forest (RF)	12	2.22 (1.18–3.25)	3.22	1.80	98.5	718.2	<0.001
Support vector machine (SVM)	9	1.68 (1.34–2.02)	0.23	0.48	93.8	129.2	<0.001
XGBoost	12	1.75 (1.32–2.19)	0.55	0.74	98.4	686.8	<0.001
Decision tree (DT)	7	0.73 (0.31–1.14)	0.28	0.53	96.3	161.2	<0.001
Artificial neural network (ANN)	3	1.67 (0.65–2.69)	0.78	0.88	95.4	43.1	<0.001
AdaBoost	4	1.57 (1.35–1.79)	0.04	0.21	86.2	21.7	<0.001
Gradient boosting DT (GBDT)	3	1.91 (0.63–3.19)	1.27	1.13	99.2	240.3	<0.001
Naïve Bayes (NB)	2	1.32 (1.22–1.42)	<0.01	0.02	2.8	1.0	0.31
k-nearest neighbor (k-NN)	2	1.75 (1.31–2.19)	0.09	0.31	93.0	14.4	<0.001
LightGBM	5	1.17 (0.50-1.83)	0.53	0.73	99.1	433.8	<0.001

Test for subgroup differences: Q = 26.27, df = 10, p = 0.003

Table S7. Summary statistics of Pooled AUC meta-analysis (training cohort)

ML architecture	No. of models (k)	Pooled logit AUC (95% CI)	τ^2	τ	I^2 (%)	Q statistic	p for heterogeneity
All models	162	1.17 (1.10-1.24)	0.17	0.41	98.7	12153.2	<0.001
LR	36	1.12 (1.03- 1.20)	0.05	0.23	98.0	1726.0	<0.001

RF	31	1.30 (1.15-1.45)	0.14	0.38	90.6	317.6	<0.001
SVM	19	1.17 (0.99- 1.36)	0.13	0.36	91.8	218.7	<0.001
XGBoost	23	1.33 (1.16-1.50)	0.15	0.38	96.9	721	<0.001
LightgBM	10	1.24 (0.89-1.58)	0.30	0.54	95.6	204	<0.001
NB	3	1.10 (0.31-1.89)	0.46	0.68	96.9	64.6	<0.001
Softmax	3	1.27 (0.75-1.80)	0.19	0.44	95.4	43.4	<0.001
Ensemble	3	1.33 (1.22-1.44)	<0.01	<0.01	34.8	3.1	0.21
k-NN	7	0.65 (0.46-0.85)	0.04	0.21	69.1	19.5	0.003
DT	11	0.79 (0.52- 1.06)	0.19	0.43	91.2	113.1	<0.001
GBDT	6	1.30 90.86-1.75)	0.27	0.52	90.6	53.2	<0.001
AdaBoost	6	1.13 (0.74-1.52)	0.20	0.45	94.8	96.8	<0.001
ANN	4	1.21 (0.32-2.10)	0.78	0.89	97.4	115.5	<0.001

Test for subgroup differences: Q = 51.37, df = 12, $p < 0.001$

Pooled estimates are reported on the logit AUC scale. Meta-analyses were performed using random-effects models with restricted maximum likelihood (REML) estimation. Between-study heterogeneity was quantified using τ^2, τ, and I^2 statistics.

Table S8. Summary statistics from the bivariate random effect meta-analysis of sensitivity and specificity of ML models

Metric	Training cohort	Validation cohort
Meta-analysis model	Bivariate random-effects (Reitsma)	Bivariate random-effects (Reitsma)
Estimation method	REML	REML
Pooled sensitivity (95% CI)	0.75 (0.71–0.79)	0.61 (0.53–0.69)
Pooled specificity (95% CI)	0.81 (0.72–0.87)	0.82 (0.77–0.86)*
False positive rate (95% CI)	0.19 (0.13–0.29)	0.18 (0.14–0.23)
Between-study SD (τ) – Sensitivity	0.42	1.44
Between-study SD (τ) – False positive rate	1.04	1.27
Correlation (Sensitivity vs FPR)	0.47	0.91
Area under the ROC curve (AUC)	0.815	0.795
Partial AUC (normalized)	0.789	0.727
I^2 (Zhou–Dendukuri)	38.8%	12.1%
Log-likelihood	27.53	89.23
AIC	−45.05	−168.46
BIC	−37.42	−153.61

Table S9. GRADE assessment of certainty of evidence

**Outcome**	**No. of Models (Design)**	**Pooled Estimate (95% CI)**	**Risk of Bias**	**Inconsistency**	**Indirectness**	**Imprecision**	**Overall Certainty**
**Validation AUC**	162 (Observational)[1]	0.76 (0.75–0.78)	Serious[2]	Very Serious[3]	Not serious	Not serious	**VERY LOW** [4]
**Training AUC**	81 (Observational)[1]	0.83 (0.79–0.85)	Serious[2]	Very Serious[3]	Serious[5]	Not serious	**VERY LOW** [6]
**Validation Sensitivity**	72 (Observational)[1]	0.61 (0.53–0.69)	Serious[2]	Serious[7]	Not serious	Serious[8]	**VERY LOW** [9]
**Validation Specificity**	72 (Observational)[1]	0.82 (0.77–0.86)	Serious[2]	Serious[7]	Not serious	Serious[8]	**VERY LOW** [10]
**Training Sensitivity**	17 (Observational)[1]	0.75 (0.71–0.79)	Serious[2]	Serious[7]	Serious[5]	Not serious	**VERY LOW** [11]
**Training Specificity**	17 (Observational)[1]	0.81 (0.72–0.87)	Serious[2]	Serious[7]	Serious[5]	Serious[8]	**VERY LOW** [12]

[1]**Study Design:** Predominantly retrospective cohort studies, with a small number of prospective and post-hoc RCT cohorts.

[2]**Risk of Bias (RoB):** Downgraded one level; a significant portion of models (44.4%) were at high risk of bias per PROBAST criteria, mainly in the analysis and outcome domains.

[3]**Inconsistency (AUC):** Downgraded two levels for very serious inconsistency; extreme statistical heterogeneity was observed ($I^2 > 98\%$).

[4]**Certainty (Validation AUC):** Started at LOW (observational). Downgraded 1 (RoB) and 2 (Inconsistency). Final: **VERY LOW**.

[5]**Indirectness (Training AUC):** Downgraded one level; training cohorts reflect model development/optimization rather than independent clinical validation.

[6]**Certainty (Training AUC):** Started at LOW. Downgraded 1 (RoB), 2 (Inconsistency), and 1 (Indirectness). Final: **VERY LOW**.

[7]**Inconsistency (Sensitivity/Specificity):** Downgraded one level; I^2 ranged from 82–95% among diagnostic test accuracy metrics.

[8]**Imprecision:** Downgraded one level due to wide confidence intervals affecting clinical certainty.

[9]**Certainty (Validation Sensitivity):** Started at LOW, downgraded 1 for RoB, 1 for inconsistency → **VERY LOW**.

[10]**Certainty (Validation Specificity):** Started at LOW. Downgraded 2 levels (RoB, Inconsistency). Final: **VERY LOW**.

[11]**Certainty (Training Sensitivity):** Started at LOW, downgraded 1 for RoB, 1 for inconsistency, 1 for indirectness → **VERY LOW**.

[12]**Certainty (Training Specificity):** Started at LOW, downgraded 1 for RoB, 1 for inconsistency, 1 for indirectness, 1 for imprecision → **VERY LOW**.

Table S10. Top predictors ranked by feature importance identified in the included studies

Study	Predictors
Yang Li et al (2020)	Gender<br>LVEF<br>Preoperative serum creatinine<br>Serum uric Acid<br>Platelet
Guy Lei et al (2020)	BMI<br>Preoperative total Bilirubin<br>Preoperative serum creatinine<br>Preoperative hemoglobin<br>Surgery time
Tseng et al (2020)	Intraoperative urine output<br>Intraoperative RBCs transfusion<br>Intraoperative blood products<br>Preoperative serum creatinine<br>Preoperative hemoglobin
Xinsai et al (2022)	Preoperative serum creatinine<br>BUN<br>Plasma transfusion<br>Los in ICU<br>MVT
Zhang et al (2022)	Central venous pressure<br>Postoperative hemoglobin<br>Postoperative serum potassium<br>Conventional ultrafiltration<br>Postoperative LDH
Kalisnik et al (2022)	eGFR<br>Age<br>Urine output<br>Postoperative hemoglobin<br>LVEF
Li et al (2022)	D-Dimmer

	eGFR<br>Leukocyte<br>Age<br>Emergency surgery
Thongprayoon et al (2022)	Preoperative eGFR<br>Cardiac surgery type<br>Coagulopathy<br>Periphral vascular disorders<br>Hypertension
Ejmalian et al (2022)	Preoperative serum creatinine<br>Cardiopulmonary bypass time<br>Blood sugar<br>Albumin<br>Age
Petrosyan et al (2022)	CARE score<br>Preoperative eGFR<br>Surgery type<br>Age<br>NYHA class
Jia et al (2023)	Serum creatinine 24hrs after operation<br>Preoperative serum creatinine<br>Body surface area<br>Pulmonary hypertension<br>Preoperative eGFR
Yan et al (2023)	LVEF<br>BMI<br>Lactate<br>Gender<br>Hypertension
Shao et al (2023)	IVST<br>Baseline ACT<br>Left atrial diameter<br>LVEF<br>Diastolic BP
Tong et al (2023)	Creatinine clearance<br>Intraoperative urine output<br>Age<br>Cardiopulmonary bypass time<br>Sodium bicarbonate
Zheng et al (2023)	Neutrophil percentage preoperative<br>Eosinophil preoperative<br>Monocyte preoperative<br>Emergency postoperative potassium concentration<br>Low density lipoprotein preoperative
Li et al (2023)	Prothrombin time<br>BUN<br>Serum creatinine<br>Platelets<br>Albumin
Jiang et al (2023)	Preoperative serum creatinine<br>Assisted ventilation<br>Lipid lowering agents<br>IV Nitro-glycerine<br>Hyperlipidaemia
Fan et al (2023)	H-FABP

	N terminal -pro brain natriuretic peptide<br>Uric acid<br>Soluble ST2<br>LDH
Dai et al (2023)	Intraoperative urine output<br>OUT CPB MAP 65 time<br>Blood transfusion<br>Smoking<br>Blood transfusion
Gao et al (2023)	eGFR postoperative<br>Postoperative creatinine<br>eGFR preoperative<br>Surgical time<br>Postoperative NT proBNP
Zeng et al (2024)	Intraoperative urine output<br>Intraoperative Sufantanil dosage<br>Intraoperative dexmedetomidine dosage<br>Mean arterial pressure I (induction period)<br>Intraoperative hypotension duration
Liu et al (2024)	Serum cystatin C<br>APACHE II<br>Procalcitonin postoperative<br>Aspartate transaminase postoperative<br>Platelet postoperative
Chango Han et al. 2025	Perfusion Pressure<br>Euroscore II<br>Age<br>Hemoglobin<br>Albumin
Song et al. 2025	Use of insulin aspart<br>Acarbose<br>Spironolactone<br>Alfentanil<br>Dezocine
XinPei et al. 2025	Valve replacement<br>Pre-operative Hypertension<br>Large vegetations<br>NYHA functional class<br>Alcoholism
Zhang et al. 2025	eGFR<br>Epinephrine<br>Calcium<br>Hypertension<br>Hypoglycemic drugs
Kuroush Nezafati et al. 2025	Race<br>Type of Surgery<br>Hypertension

	Systolic BP ICU<br>hemoglobin
Haiming Li et al.	Baseline eGFR<br>Intraoperative epinephrine<br>CPB<br>Surgery time<br>Intraoperative IABP
Biao Hou	Calcium<br>Neutrophil<br>HDL-C<br>Direct bilirubin<br>Total protein
Zishan Li	Anchor age<br>Serum creatinine<br>BUN<br>Potassium<br>Sodium
Yang Xu	Age<br>Weight<br>Mean arterial pressure<br>Atrial fibrillation<br>Preoperative hemoglobin
Qing Sun	Intraoperative RBC blood transfusion<br>Cardiopulmonary bypass time, min<br>Preoperative hemoglobin<br>BMI<br>Hypertension
Zheyuan Chen	Ventillation time<br>Urine output<br>Diuretics<br>Serum creatinine<br>Heart rate

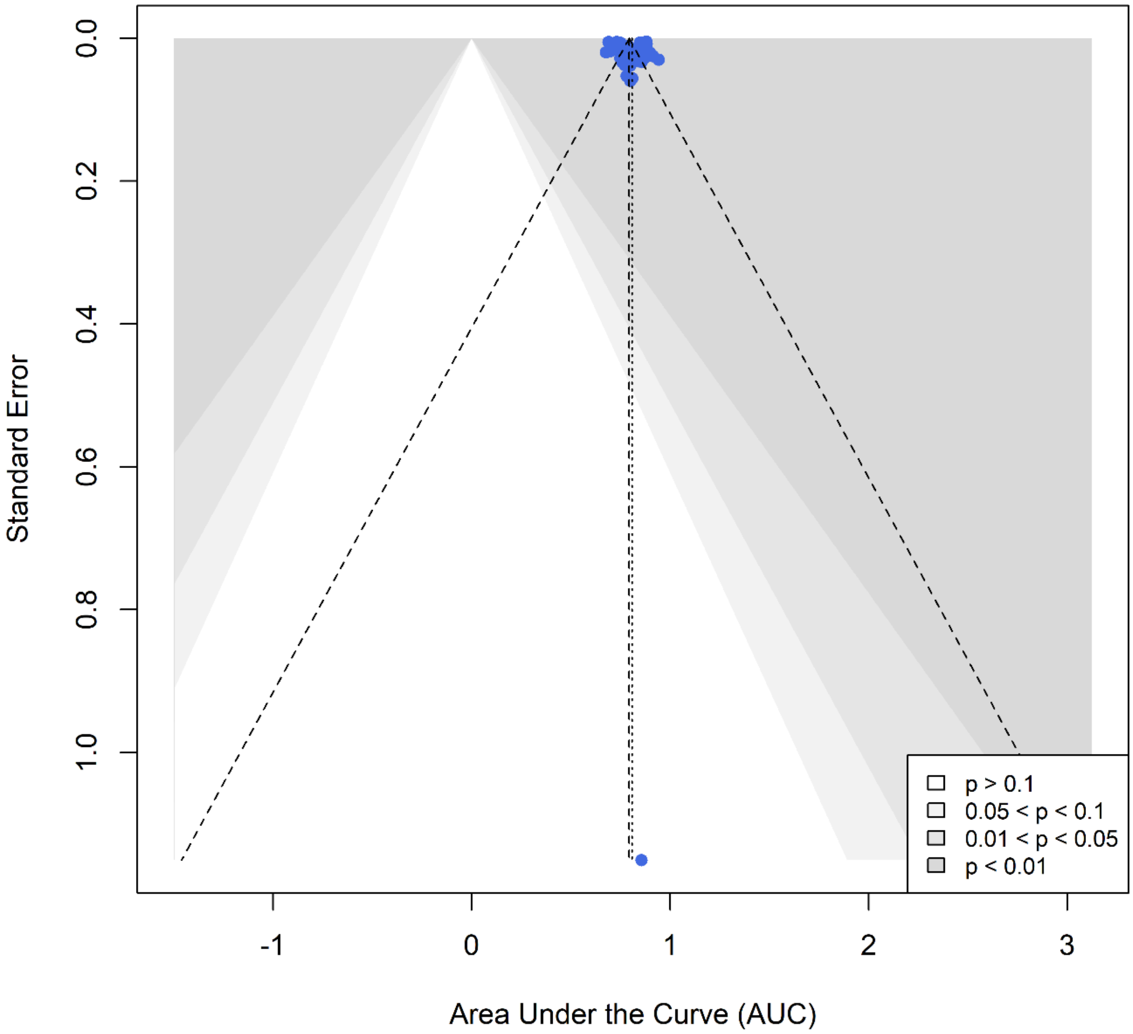

Figure S1. Funnel plot assessing publication bias for machine learning–based AUC in validated models. The funnel plot displays each study's AUC against its standard error. The vertical line represents the pooled AUC from random-effects meta-analysis (REML method, Hartung-Knapp adjustment). Contour shading indicates statistical significance levels ($p < 0.01$, 0.01–0.05, 0.05–0.1, and $p > 0.1$). Egger's regression test was performed to statistically assess asymmetry, with non-significant results suggesting minimal publication bias.

R codes

```
###########################################################################################
# Supplementary R Code
# Section 1 Geographical Distribution Heatmap of Included Studies
# Study: Machine learning–based prediction models for AKI after cardiac surgery
# Author: Zirwa Asim
###########################################################################################

# -------------------------------
# 1. Load required packages
# -------------------------------
library(ggplot2)      # For plotting
library(dplyr)        # For data manipulation
library(rnaturalearth) # For world map data
library(rnaturalearthdata)
library(sf)           # For spatial data handling

# -------------------------------
# 2. Get world map data
# -------------------------------
# 'scale = "medium"' provides a balance between detail and file size
world <- ne_countries(scale = "medium", returnclass = "sf")

# -------------------------------
# 3. Study counts per country
# -------------------------------
study_data <- data.frame(
  country = c("China", "United States of America", "Iran", "South Korea",
              "Australia", "Germany", "France", "Canada", "Taiwan"),
  studies = c(35, 3, 1, 1, 1, 1, 1, 1, 1)
)

# -------------------------------
# 4. Merge map with study data
```

```
# -------------------------------
world_data <- world %>%
  left_join(study_data, by = c("name" = "country"))

# -------------------------------
# 5. Compute centroids for country labels
# Only for countries with studies
# -------------------------------
label_data <- world_data %>%
  filter(!is.na(studies)) %>%
  st_centroid()  # Computes central coordinates for label placement

# -------------------------------
# 6. Create the heatmap
# -------------------------------
heatmap_plot <- ggplot(world_data) +
  geom_sf(aes(fill = studies), color = "black", size = 0.2) +  # Countries colored by study count

  # Country labels
  geom_sf_text(
    data = label_data,
    aes(label = name),
    size = 3,
    fontface = "bold",
    color = "black"
  ) +

  # Color gradient: low = light green, high = red
  scale_fill_gradient(
    name = "Number of Studies",
    low = "lightgreen",
    high = "red",
    na.value = "grey90"
  ) +
```

```
  theme_minimal() +
  theme(
    legend.position = "right",
    panel.grid.major = element_line(color = "grey80", size = 0.2),
    axis.text = element_blank(),
    axis.title = element_blank(),
    panel.background = element_rect(fill = "white", color = NA)
  )

# Display the plot in R
print(heatmap_plot)

# -------------------------------
# 7. Save high-resolution TIFF
# -------------------------------
ggsave(
  filename = "geographic_distribution_with_country_names.tiff",
  plot = heatmap_plot,
  width = 10,
  height = 5,
  dpi = 600,
  compression = "lzw"
)
##########################################################################################
# End of Section 1
```

```
##########################################################################################
# Section 2: PROBAST Risk-of-Bias Analysis
##########################################################################################
# -------------------------------
# 1. Load required packages
# -------------------------------
library(readxl)   # For reading Excel files
library(dplyr)    # For data manipulation
```

```
library(tidyr)     # For reshaping data
library(ggplot2)   # For plotting
library(scales)    # For percentage labels

# -------------------------------
# 2. Read dataset
# -------------------------------
# The Excel file should contain columns for each PROBAST domain:
# Participants, Predictors, Outcome, Analysis, Overall
data <- read_excel("Book1.xlsx")

# -------------------------------
# 3. Define PROBAST domains
# -------------------------------
rob_domains <- c(
  "Participants",
  "Predictors",
  "Outcome",
  "Analysis",
  "Overall"
)

# -------------------------------
# 4. Reshape data to long format and calculate percentages
# -------------------------------
result_long <- data %>%
  select(any_of(rob_domains)) %>%   # Select only domain columns
  pivot_longer(
    cols = everything(),
    names_to = "Domain",
    values_to = "Risk"
  ) %>%
  count(Domain, Risk) %>%
  group_by(Domain) %>%
```

```
  mutate(Percentage = n / sum(n)) %>%
  ungroup()

# Check intermediate result
print(head(result_long))

# -------------------------------
# 5. Order domains and risk categories
# -------------------------------
# Order domains
result_long$Domain <- factor(
  result_long$Domain,
  levels = rob_domains,
  labels = c("Participants", "Predictors", "Outcomes", "Analysis", "Overall")
)

# Order risk levels
result_long$Risk <- factor(
  result_long$Risk,
  levels = c("High", "Low", "Unclear")
)

# -------------------------------
# 6. Plot PROBAST risk-of-bias bar chart
# -------------------------------
rob_plot <- ggplot(result_long,
                   aes(x = Domain, y = Percentage, fill = Risk)) +
  geom_bar(stat = "identity", width = 0.7) +
  coord_flip() +
  scale_y_continuous(labels = percent, expand = c(0, 0)) +
  scale_fill_manual(
    values = c(
      "Low" = "#00FF00",       # Green for low risk
      "Unclear" = "#FFFF00",   # Yellow for unclear
```

```
    "High" = "#FF0000"      # Red for high risk
   )
  ) +
  labs(
   x = "",
   y = "Percentage"
  ) +
  theme_minimal(base_size = 14) +
  theme(
   legend.position = "bottom",
   panel.grid.major.y = element_blank(),
   plot.margin = margin(10, 40, 10, 10)
  )

# Display the plot
print(rob_plot)

# -------------------------------
# 7. Save high-resolution TIFF for publication
# -------------------------------
ggsave(
  filename = "Risk_of_Bias_PROBAST.tiff",
  plot = rob_plot,
  width = 12,
  height = 6,
  dpi = 600,
  compression = "lzw"
)

# -------------------------------
# 8. PROBAST domain-wise summary table
# -------------------------------
rob_domain_summary <- result_long %>%
  mutate(
```

```
    Percentage = round(Percentage * 100, 1)
  ) %>%
  select(Domain, Risk, Percentage) %>%
  group_by(Domain, Risk) %>%
  summarise(
    Percentage = unique(Percentage),
    .groups = "drop"
  ) %>%
  pivot_wider(
    names_from = Risk,
    values_from = Percentage,
    values_fill = 0
  ) %>%
  arrange(Domain)

# Print domain-wise summary
print(rob_domain_summary)

##########################################################################################
# End of Section 2: PROBAST Risk-of-Bias Analysis
```

```
##########################################################################################
# Supplementary R Code
# Section 3: Random-Effects Meta-Analysis of AUC (with Heterogeneity)
##########################################################################################

# -------------------------------
# 1. Load Required Packages
# -------------------------------
library(readxl)      # Read Excel files
library(dplyr)       # Data manipulation
library(meta)        # Meta-analysis
library(forestplot)  # Forest plots
library(grid)        # Grid graphics
library(magick)      # Image manipulation for panel figures
```

```
# --------------------------------
# 2. Function: Run Random-Effects Meta-Analysis
# --------------------------------
run_model_meta <- function(file_name) {
  # 2.1 Load data
  data <- read_excel(paste0(file_name, ".xlsx"))

  # 2.2 Prepare effect sizes (logit transformation)
  data_es <- data %>%
    mutate(
      AUC_adj = pmin(pmax(AUC, 0.001), 0.999),        # Avoid 0 or 1
      CI_L_adj = pmin(pmax(`CI Lower`, 0.001), 0.999),
      CI_U_adj = pmin(pmax(`CI Upper`, 0.001), 0.999),
      TE = log(AUC_adj / (1 - AUC_adj)),
      seTE = (log(CI_U_adj / (1 - CI_U_adj)) - log(CI_L_adj / (1 - CI_L_adj))) / (2 * 1.96)
    )

  # 2.3 Keep only models with ≥ 2 studies
  valid_models <- data_es %>% count(`ML models`) %>% filter(n >= 2) %>% pull(`ML models`)
  data_es <- data_es %>% filter(`ML models` %in% valid_models)

  # 2.4 Random-effects meta-analysis (logit scale)
  meta_auc <- metagen(
    TE = TE,
    seTE = seTE,
    subgroup = `ML models`,
    data = data_es,
    sm = "logit",
    common = FALSE,
    random = TRUE,
    method.tau = "REML"
  )
```

```
# -------------------------------
# 2.5 Extract Model-Specific Results
# -------------------------------
model_results <- data.frame(
  Model = names(meta_auc$TE.random.w),
  AUC   = plogis(meta_auc$TE.random.w),
  Lower = plogis(meta_auc$lower.random.w),
  Upper = plogis(meta_auc$upper.random.w),
  N     = meta_auc$k.w
)

# -------------------------------
# 2.6 Overall Summary
# -------------------------------
overall <- data.frame(
  Model = "Summary",
  AUC   = plogis(meta_auc$TE.random),
  Lower = plogis(meta_auc$lower.random),
  Upper = plogis(meta_auc$upper.random),
  N     = meta_auc$k
)

# -------------------------------
# 2.7 Heterogeneity Statistics
# -------------------------------
heterogeneity <- data.frame(
  Tau2 = meta_auc$tau^2,
  I2   = meta_auc$I2,
  Q    = meta_auc$Q,
  df   = meta_auc$df.Q,
  pval = meta_auc$pval.Q
)

# Return results and heterogeneity
```

```
  return(list(
    results = rbind(model_results, overall),
    heterogeneity = heterogeneity
  ))
}

# -------------------------------
# 3. Run Meta-Analysis on Training and Validation Sets
# -------------------------------
training_meta   <- run_model_meta("training_AUC_R")
validation_meta <- run_model_meta("validation_AUC_R")

training_results   <- training_meta$results
validation_results <- validation_meta$results

# Print Heterogeneity
cat("\n===== Training Set Heterogeneity =====\n")
print(training_meta$heterogeneity)

cat("\n===== Validation Set Heterogeneity =====\n")
print(validation_meta$heterogeneity)

# -------------------------------
# 4. Function: Save High-Resolution Forest Plot as TIFF
# -------------------------------
save_forestplot_tiff <- function(plot_data, filename) {
  # Close any open graphics devices
  while (!is.null(dev.list())) dev.off()

  # Create table text for forest plot
  tabletext <- cbind(
    c("Model", as.character(plot_data$Model)),
    c("No. of Studies (N)", as.character(plot_data$N)),
    c("AUC (95% CI)", sprintf("%.2f (%.2f–%.2f)", plot_data$AUC, plot_data$Lower, plot_data$Upper))
```

```
  )

  # Open TIFF device (600 DPI)
  tiff(filename, width = 6000, height = 4000, res = 600, compression = "lzw")
  grid.newpage()

  # Generate forest plot
  p <- forestplot(
    labeltext = tabletext,
    mean  = c(NA, plot_data$AUC),
    lower = c(NA, plot_data$Lower),
    upper = c(NA, plot_data$Upper),
    is.summary = c(TRUE, rep(FALSE, nrow(plot_data) - 1), TRUE),
    zero = 0.5,
    clip = c(0, 1),
    xticks = seq(0, 1, 0.2),
    boxsize = 0.2,
    lwd.ci = 3,
    lwd.zero = 2,
    col = fpColors(box = "blue", line = "red", summary = "darkgreen"),
    txt_gp = fpTxtGp(label = gpar(cex = 0.9), ticks = gpar(cex = 0.8), xlab = gpar(cex = 1.0)),
    xlab = "Area Under the Curve (AUC)",
    new_page = FALSE
  )

  print(p)
  dev.off()
}

# Save plots
save_forestplot_tiff(training_results, "train_auc_600.tiff")
save_forestplot_tiff(validation_results, "val_auc_600.tiff")

# -------------------------------
```

```
# 5. Combine Training & Validation Plots into Panel Figure
# --------------------------------
train_img <- image_read("train_auc_600.tiff")
val_img   <- image_read("val_auc_600.tiff")

# White padding for labels
pad_white <- image_blank(width = image_info(train_img)$width, height = 150, color = "white")

# Annotate with (A) and (B)
train_labeled <- image_annotate(
  image_append(c(pad_white, train_img), stack = TRUE),
  "(A) Training Cohort",
  size = 180, weight = 700, gravity = "northwest", location = "+50+30"
)

val_labeled <- image_annotate(
  image_append(c(pad_white, val_img), stack = TRUE),
  "(B) Validation Cohort",
  size = 180, weight = 700, gravity = "northwest", location = "+50+30"
)

# Combine vertically
final_panel <- image_append(c(train_labeled, val_labeled), stack = TRUE)

# Save final high-resolution panel figure
image_write(final_panel, "Final_Figure_AUC_600DPI.tiff", density = 600, compression = "lzw")

cat("\n✅ Success: Training & Validation Panel saved as 'Final_Figure_AUC_600DPI.tiff'")
#######################################################################################
# End of Section 3################################
```

```
#############################################################
### SECTION 4: Model-wise Sensitivity & Specificity Table
#############################################################
```

```
library(dplyr)

# Function to extract model-wise bivariate estimates
extract_modelwise_biv <- function(df) {

  df %>%
    group_by(`Model types`) %>%
    summarise(
      TP = sum(round(Sensitivity * Events), na.rm = TRUE),
      FN = sum(Events - round(Sensitivity * Events), na.rm = TRUE),
      TN = sum(round(Specificity * (Total_N - Events)), na.rm = TRUE),
      FP = sum((Total_N - Events) - round(Specificity * (Total_N - Events)), na.rm = TRUE),
      n_studies = n(),
      .groups = "drop"
    ) %>%
    rowwise() %>%
    mutate(
      # Fit bivariate Reitsma model for each model type
      fit = list(reitsma(data.frame(
        TP = TP, FN = FN, FP = FP, TN = TN
      ), calculate_TP="TP", calculate_FP="FP", calculate_FN="FN", calculate_TN="TN")),
      summary_fit = list(summary(fit))
    ) %>%
    mutate(
      sens_pooled = invlogit(summary_fit$coefficients[1,1]),
      sens_lower  = invlogit(summary_fit$coefficients[1,5]),
      sens_upper  = invlogit(summary_fit$coefficients[1,6]),
      spec_pooled = 1 - invlogit(summary_fit$coefficients[2,1]),
      spec_lower  = 1 - invlogit(summary_fit$coefficients[2,6]),
      spec_upper  = 1 - invlogit(summary_fit$coefficients[2,5])
    ) %>%
    select(`Model types`, n_studies,
           sens_pooled, sens_lower, sens_upper,
```

```
      spec_pooled, spec_lower, spec_upper)
}

# Run for training and validation sets
train_modelwise <- extract_modelwise_biv(training_data)
val_modelwise   <- extract_modelwise_biv(validation_data)

# Optional: Add overall pooled row
overall_train <- data.frame(
  `Model types` = "Overall",
  n_studies = sum(train_modelwise$n_studies),
  sens_pooled = pooled_train$sens$pooled,
  sens_lower  = pooled_train$sens$lower,
  sens_upper  = pooled_train$sens$upper,
  spec_pooled = pooled_train$spec$pooled,
  spec_lower  = pooled_train$spec$lower,
  spec_upper  = pooled_train$spec$upper
)

overall_val <- data.frame(
  `Model types` = "Overall",
  n_studies = sum(val_modelwise$n_studies),
  sens_pooled = pooled_val$sens$pooled,
  sens_lower  = pooled_val$sens$lower,
  sens_upper  = pooled_val$sens$upper,
  spec_pooled = pooled_val$spec$pooled,
  spec_lower  = pooled_val$spec$lower,
  spec_upper  = pooled_val$spec$upper
)

# Bind together
train_modelwise <- rbind(train_modelwise, overall_train)
val_modelwise   <- rbind(val_modelwise, overall_val)
```

```
# Print tables for supplementary material
cat("\n===== Training Cohort Model-wise Sensitivity & Specificity =====\n")
print(train_modelwise)

cat("\n===== Validation Cohort Model-wise Sensitivity & Specificity =====\n")
print(val_modelwise)
##########################################################################################
########End of section 4###################################################################
```

```
############################################################
### SECTION 5: Publication Bias Analysis (AUC)
############################################################

# 1. Load libraries
library(readxl)
library(meta)
library(dplyr)

# 2. Read dataset
pb_data <- read_excel("publicationbias.xlsx")

# 3. Compute standard error from 95% CI
# Ensure CI_Upper and CI_Lower are numeric
pb_data <- pb_data %>%
  mutate(SE = (CI_Upper - CI_Lower) / (2 * 1.96))

# 4. Random-effects meta-analysis of AUC
# Hartung-Knapp adjustment recommended for DTA meta-analysis
m_auc <- metagen(
  TE = AUC,          # Effect estimate (AUC)
  seTE = SE,         # Standard error
  studlab = `Study ID`,
  data = pb_data,
  sm = "AUC",
  method.tau = "REML",
```

```
  hakn = TRUE
)

# 5. Egger's regression test for funnel plot asymmetry
egg_test <- metabias(m_auc, method.bias = "linreg")
cat("===== Egger's Regression Test for Publication Bias =====\n")
print(egg_test)

# 6. Generate publication-quality funnel plot (interactive)
funnel(m_auc,
       xlab = "Area Under the Curve (AUC)",
       ylab = "Standard Error",
       pch = 16,                # Solid points
       col = "royalblue",       # Professional color
       contour = c(0.9, 0.95, 0.99),  # Significance contours
       col.contour = c("gray95", "gray90", "gray85"))
legend("bottomright",
       legend = c("p > 0.1", "0.05 < p < 0.1", "0.01 < p < 0.05", "p < 0.01"),
       fill = c("white", "gray95", "gray90", "gray85"),
       cex = 0.8)

# 7. Save high-resolution funnel plot as TIFF for publication
tiff(
  filename = "Figure_S1_Funnel_Plot_AUC.tiff",
  width = 7, height = 7, units = "in",
  res = 600, compression = "lzw"
)

funnel(m_auc,
       xlab = "Area Under the Curve (AUC)",
       ylab = "Standard Error",
       pch = 16,
       col = "royalblue",
       contour = c(0.9, 0.95, 0.99),
```

```
      col.contour = c("gray95", "gray90", "gray85"))
legend("bottomright",
      legend = c("p > 0.1", "0.05 < p < 0.1", "0.01 < p < 0.05", "p < 0.01"),
      fill = c("white", "gray95", "gray90", "gray85"),
      cex = 0.8)

dev.off()

cat("\n===== Funnel plot saved as 'Figure_S1_Funnel_Plot_AUC.tiff' =====\n")

#############################################################################################
##############################End of section 5############################################
################################################################
### SECTION 6: Predictor Frequency Plot
################################################################

# 1. Load required libraries
library(readxl)
library(ggplot2)
library(dplyr)

# 2. Read predictor data
# Expected columns in Excel: Predictors | Number of studies
predictor_data <- read_excel("Predictors.xlsx", sheet = "Predictors")

# 3. Clean and rename columns
predictor_data <- predictor_data %>%
  rename(
    Predictor = Predictors,
    Count = `Number of studies`
  )

# 4. Convert Count to factor for color mapping
predictor_data <- predictor_data %>%
```

```
  mutate(Count_factor = factor(Count))

# 5. Create horizontal bar plot
predictor_plot <- ggplot(
  predictor_data,
  aes(
    x = reorder(Predictor, Count),  # Reorder by count
    y = Count,
    fill = Count_factor
  )
) +
  geom_bar(stat = "identity", width = 0.8) +
  coord_flip() +                    # Horizontal bars
  labs(
    x = "Predictor",
    y = "Number of studies",
    fill = "Frequency"
  ) +
  theme_minimal(base_size = 12) +
  theme(
    plot.title = element_blank(),       # No title
    legend.position = "right",
    panel.grid.major.y = element_blank(),
    axis.text.y = element_text(size = 11),
    axis.text.x = element_text(size = 11)
  ) +
  scale_fill_brewer(palette = "Blues")  # Nice professional palette

# 6. Save high-resolution TIFF for publication
ggsave(
  filename = "ML_predictor_frequency.tiff",
  plot = predictor_plot,
  device = "tiff",
  dpi = 600,
```

```
  width = 8,
  height = 10,
  units = "in",
  compression = "lzw"
)

#######################################################################################
######################End of section 6####################################################
#######################################################################################
```
